# Large-Scale Genome-Wide Association Studies and Meta-Analyses of Longitudinal Change in Adult Lung Function

**DOI:** 10.1371/journal.pone.0100776

**Published:** 2014-07-01

**Authors:** Wenbo Tang, Matthew Kowgier, Daan W. Loth, María Soler Artigas, Bonnie R. Joubert, Emily Hodge, Sina A. Gharib, Albert V. Smith, Ingo Ruczinski, Vilmundur Gudnason, Rasika A. Mathias, Tamara B. Harris, Nadia N. Hansel, Lenore J. Launer, Kathleen C. Barnes, Joyanna G. Hansen, Eva Albrecht, Melinda C. Aldrich, Michael Allerhand, R. Graham Barr, Guy G. Brusselle, David J. Couper, Ivan Curjuric, Gail Davies, Ian J. Deary, Josée Dupuis, Tove Fall, Millennia Foy, Nora Franceschini, Wei Gao, Sven Gläser, Xiangjun Gu, Dana B. Hancock, Joachim Heinrich, Albert Hofman, Medea Imboden, Erik Ingelsson, Alan James, Stefan Karrasch, Beate Koch, Stephen B. Kritchevsky, Ashish Kumar, Lies Lahousse, Guo Li, Lars Lind, Cecilia Lindgren, Yongmei Liu, Kurt Lohman, Thomas Lumley, Wendy L. McArdle, Bernd Meibohm, Andrew P. Morris, Alanna C. Morrison, Bill Musk, Kari E. North, Lyle J. Palmer, Nicole M. Probst-Hensch, Bruce M. Psaty, Fernando Rivadeneira, Jerome I. Rotter, Holger Schulz, Lewis J. Smith, Akshay Sood, John M. Starr, David P. Strachan, Alexander Teumer, André G. Uitterlinden, Henry Völzke, Arend Voorman, Louise V. Wain, Martin T. Wells, Jemma B. Wilk, O. Dale Williams, Susan R. Heckbert, Bruno H. Stricker, Stephanie J. London, Myriam Fornage, Martin D. Tobin, George T. O′Connor, Ian P. Hall, Patricia A. Cassano

**Affiliations:** 1 Division of Nutritional Sciences, Cornell University, Ithaca, New York, United States of America; 2 Ontario Institute for Cancer Research and Biostatistics Division, Dalla Lana School of Public Health, University of Toronto, Toronto, Ontario, Canada; 3 Department of Epidemiology, Erasmus Medical Center, Rotterdam, the Netherlands; 4 Netherlands Healthcare Inspectorate, The Hague, the Netherlands; 5 University of Leicester, Genetic Epidemiology Group, Department of Health Sciences, Leicester, United Kingdom; 6 National Institute for Health Research (NIHR) Leicester Respiratory Biomedical Research Unit, Glenfield Hospital, Leicester, United Kingdom; 7 Epidemiology Branch, National Institute of Environmental Health Sciences, National Institutes of Health, U.S. Department of Health and Human Services, Research Triangle Park, North Carolina, United States of America; 8 Division of Respiratory Medicine, University Hospital of Nottingham, Nottingham, United Kingdom; 9 Computational Medicine Core, Center for Lung Biology, Division of Pulmonary & Critical Care Medicine, Department of Medicine, University of Washington, Seattle, Washington, United States of America; 10 Icelandic Heart Association, Kopavogur, Iceland; 11 University of Iceland, Reykjavik, Iceland; 12 Department of Biostatistics, Bloomberg School of Public Health, Johns Hopkins University, Baltimore, Maryland, United States of America; 13 Department of Medicine, School of Medicine, Johns Hopkins University, Baltimore, Maryland, United States of America; 14 Laboratory of Epidemiology, Demography, and Biometry, National Institute on Aging, National Institutes of Health, Bethesda, Maryland, United States of America; 15 Institute of Genetic Epidemiology, Helmholtz Zentrum München - German Research Center for Environmental Health, Neuherberg, Germany; 16 Department of Thoracic Surgery and Division of Epidemiology, Vanderbilt University Medical Center, Nashville, Tennessee, United States of America; 17 Centre for Cognitive Ageing and Cognitive Epidemiology, University of Edinburgh, Edinburgh, United Kingdom; 18 Division of General Medicine, Pulmonary, Allergy and Critical Care, Department of Medicine, College of Physicians and Surgeons, Columbia University, New York, New York, United States of America; 19 Department of Epidemiology, Mailman School of Public Health, Columbia University, New York, New York, United States of America; 20 Department of Respiratory Medicine, Ghent University Hospital, Ghent, Belgium; 21 Department of Respiratory Medicine, Erasmus Medical Center, Rotterdam, the Netherlands; 22 Department of Biostatistics, University of North Carolina at Chapel Hill, Chapel Hill, North Carolina, United States of America; 23 Swiss Tropical and Public Health Institute, Basel, Switzerland; 24 University of Basel, Basel, Switzerland; 25 Medical Genetics Section, University of Edinburgh Molecular Medicine Centre and MRC Institute of Genetics and Molecular Medicine, Western General Hospital, Edinburgh, United Kingdom; 26 Department of Psychology, University of Edinburgh, Edinburgh, United Kingdom; 27 Biostatistics Department, Boston University School of Public Health, Boston, Massachusetts, United States of America; 28 The National Heart, Lung, and Blood Institute's Framingham Heart Study, Framingham, Massachusetts, United States of America; 29 Department of Medical Sciences, Molecular Epidemiology and Science for Life Laboratory, Uppsala University, Uppsala, Sweden; 30 Institute of Molecular Medicine, University of Texas Health Science Center at Houston, Houston, Texas, United States of America; 31 Gillings School of Global Public Health, Department of Epidemiology, University of North Carolina at Chapel Hill, Chapel Hill, North Carolina, United States of America; 32 Department of Internal Medicine B; Pneumology, Cardiology, Intensive Care Medicine; Field of Research: Pneumology and Pneumological Epidemiology, University Medicine Greifswald, Greifswald, Germany; 33 Behavioral Health Epidemiology Program, Research Triangle Institute, Research Triangle Park, North Carolina, United States of America; 34 Institute of Epidemiology I, Helmholtz Zentrum München - German Research Center for Environmental Health, Neuherberg, Germany and Comprehensive Pneumology Center Munich (CPC-M), Member of the German Center for Lung Research, Munich, Germany; 35 Netherlands Consortium for Healthy Aging, Rotterdam, the Netherlands; 36 Wellcome Trust Centre for Human Genetics, University of Oxford, Oxford, UK and Department of Biostatistics, University of Liverpool, Liverpool, United Kingdom; 37 School of Medicine and Pharmacology, University of Western Australia, Perth, Western Australia, Australia; 38 Institute and Outpatient Clinic for Occupational, Social and Environmental Medicine, Ludwig-Maximilians-Universität, Munich, Germany; 39 Institute of General Practice, University Hospital Klinikum rechts der Isar, Technische Universität München, Munich, Germany; 40 Institute of Epidemiology I, Helmholtz Zentrum München - German Research Center for Environmental Health, Neuherberg, Germany; 41 Sticht Center on Aging, Wake Forest School of Medicine, Winston-Salem, North Carolina, United States of America; 42 Cardiovascular Health Research Unit, University of Washington, Seattle, Washington, United States of America; 43 Department of Medical Sciences, Uppsala University, Uppsala, Sweden; 44 Broad Institute of MIT and Harvard, Cambridge, Massachusetts, United States of America; 45 Department of Epidemiology and Prevention, Division of Public Health Sciences, Wake Forest School of Medicine, Winston-Salem, North Carolina, United States of America; 46 Department of Biostatistical Sciences, Division of Public Health Sciences, Wake Forest School of Medicine, Winston-Salem, North Carolina, United States of America; 47 Department of Statistics, University of Auckland, Auckland, New Zealand; 48 School of Social and Community Medicine, University of Bristol, Bristol, United Kingdom; 49 College of Pharmacy, University of Tennessee Health Science Center, Memphis, Tennessee, United States of America; 50 Human Genetics Center, School of Public Health, University of Texas Health Science Center at Houston, Houston, Texas, United States of America; 51 Epidemiology and Obstetrics & Gynaecology, University of Toronto, Toronto, Ontario, Canada; 52 Samuel Lunenfeld Research Institute, Toronto, Ontario, Canada; 53 Department of Epidemiology, University of Washington, Seattle, Washington, United States of America; 54 Group Health Research Institute, Group Health Cooperative, Seattle, Washington, United States of America; 55 Department of Medicine, University of Washington, Seattle, Washington, United States of America; 56 Department of Internal Medicine, Erasmus Medical Center, Rotterdam, the Netherlands; 57 Institute for Translational Genomics and Population Sciences, Los Angeles Biomedical Research Institute and Department of Pediatrics at Harbor-UCLA Medical Center, Torrance, California, United States of America; 58 Northwestern University Feinberg School of Medicine, Chicago, Illinois, United States of America; 59 University of New Mexico, Albuquerque, New Mexico, United States of America; 60 Alzheimer Scotland Dementia Research Centre, University of Edinburgh, Edinburgh, United Kingdom; 61 Division of Population Health Sciences and Education, St George's, University of London, London, United Kingdom; 62 Department for Genetics and Functional Genomics, Interfaculty Institute for Genetics and Functional Genomics, University Medicine Greifswald, Greifswald, Germany; 63 Institute for Community Medicine, Study of Health In Pomerania (SHIP)/Clinical Epidemiological Research, University Medicine Greifswald, Greifswald, Germany; 64 Department of Biostatistics, University of Washington, Seattle, Washington, United States of America; 65 Department of Statistical Science, Cornell University, Ithaca, New York, United States of America; 66 Division of Aging, Department of Medicine, Brigham and Women's Hospital and Harvard Medical School, Boston, Massachusetts, United States of America; 67 Florida International University, Miami, Florida, United States of America; 68 Section of Pulmonary, Allergy, and Critical Care Medicine, Department of Medicine, Boston University School of Medicine, Boston, Massachusetts, United States of America; 69 Department of Health Care Policy and Research, Division of Biostatistics and Epidemiology, Weill Cornell Medical College, New York, New York, United States of America; The University of Chicago, United States of America

## Abstract

**Background:**

Genome-wide association studies (GWAS) have identified numerous loci influencing cross-sectional lung function, but less is known about genes influencing longitudinal change in lung function.

**Methods:**

We performed GWAS of the rate of change in forced expiratory volume in the first second (FEV_1_) in 14 longitudinal, population-based cohort studies comprising 27,249 adults of European ancestry using linear mixed effects model and combined cohort-specific results using fixed effect meta-analysis to identify novel genetic loci associated with longitudinal change in lung function. Gene expression analyses were subsequently performed for identified genetic loci. As a secondary aim, we estimated the mean rate of decline in FEV_1_ by smoking pattern, irrespective of genotypes, across these 14 studies using meta-analysis.

**Results:**

The overall meta-analysis produced suggestive evidence for association at the novel *IL16/STARD5/TMC3* locus on chromosome 15 (*P*  =  5.71 × 10^-7^). In addition, meta-analysis using the five cohorts with ≥3 FEV_1_ measurements per participant identified the novel *ME3* locus on chromosome 11 (*P*  =  2.18 × 10^-8^) at genome-wide significance. Neither locus was associated with FEV_1_ decline in two additional cohort studies. We confirmed gene expression of *IL16*, *STARD5*, and *ME3* in multiple lung tissues. Publicly available microarray data confirmed differential expression of all three genes in lung samples from COPD patients compared with controls. Irrespective of genotypes, the combined estimate for FEV_1_ decline was 26.9, 29.2 and 35.7 mL/year in never, former, and persistent smokers, respectively.

**Conclusions:**

In this large-scale GWAS, we identified two novel genetic loci in association with the rate of change in FEV_1_ that harbor candidate genes with biologically plausible functional links to lung function.

## Introduction

Forced expiratory volume in the first second (FEV_1_) is a reliable spirometric parameter that reflects the physiological state of the lungs and airways. Reduced FEV_1_ relative to forced vital capacity (FVC), is a defining feature of chronic obstructive pulmonary disease (COPD), a leading cause of death globally.[1] FEV_1_ is also a predictor of morbidity and mortality in the general population.[2], [3] Lung function reaches its peak in early adulthood, followed by a plateau, and then subsequently declines. As first reported by Fletcher and Peto,[4] decline in lung function is accelerated in smokers, leading to increased risks of COPD and premature death. While cigarette smoking is a key risk factor for accelerated loss of lung function, genetic variation is hypothesized to also play an important role.[5], [6] Family and twin studies of the longitudinal change in lung function report heritability estimates between 10 and 39%.[7], [8]

Recent large-scale genome-wide association studies (GWAS) identified 26 novel loci for cross-sectional lung function,[9]–[11] demonstrating the power of GWAS with large sample size to identify common genetic variants with modest effect sizes. However, cross-sectional measurements in adults reflect the combination of maximal attained lung growth and subsequent decline. GWAS that specifically study the longitudinal change in lung function are needed to distinguish the genetic contributions to age-related decline. To date, only one population-based GWAS meta-analysis of longitudinal change in lung function has been reported.[12] Separate analyses were conducted in 1,441 asthmatic and 2,667 non-asthmatic participants; association was found at one novel locus in each analysis, though only the locus in non-asthmatics replicated.

In this study, we conducted primary GWAS of the rate of change in FEV_1_ in each of 14 population-based cohort studies from the Cohorts for Heart and Aging Research in Genomic Epidemiology (CHARGE) and SpiroMeta consortia, comprising 27,249 adult participants of European ancestry and 62,130 FEV_1_ measurements. We then performed meta-analysis of the cohort-specific results, followed up our most statistically significant associations in the AGES-Reykjavík cohort study and the Lung Health Study (LHS) for corroborative evidence, and explored the biological basis for identified associations using cell-specific gene expression studies, and expression quantitative trait loci (eQTL) look-up.

## Methods

### Study populations

All 14 cohort studies are members of the CHARGE or SpiroMeta Consortium (Table 1). The respective local Institutional Review Boards approved all study protocols, and written informed consent for genetic studies was obtained from all participants. Spirometry tests were performed at baseline and at least one follow-up time point by trained technicians and in accordance with the American Thoracic Society or European Respiratory Society recommendations (Methods S1 in File S1 for further details).[13] FEV_1_ measurements meeting acceptability criteria were included in the current study.

**Table 1:** Baseline characteristics of cohort studies included in the meta-analysis*.

Cohort:	ARIC	B58C	BHS	CARDIA	CHS	FHS	Health ABC
No. of participants	8,242	827	1,009	1,492	3,159	3,230	1,586
No. of FEV_1_ measurements	15,582	1,653	3,073	6,140	7,140	11,275	4,426
No. of FEV_1_ per person	2	2	7	5	3	5	4
Follow-up duration, yr	5.6	10	29	20.1	7.9	14.7	9.5
Males, %	46.5	48.6	41.6	46.9	39	47	52.7
Baseline age, yr	54.6 (5.7)	35.0 (0.2)	37.5 (12.8)	27.5 (2.3)	72.3 (5.4)	50.9 (10.3)	73.8 (2.8)
Baseline height, cm	168.7 (9.4)	170.1 (9.5)	168.1 (8.9)	171.2 (9.3)	164.6 (9.4)	168.4 (9.3)	166.8 (9.3)
Current smokers, %	20.2	27.1	20.9	24.8	10.8	24.6	6.4
Former smokers, %	32.6	41.5	16.5	17.3	35.7	39.8	49.9
Baseline pack-years†	25.9 (21.7)	7.5 (11.4)	8.2 (17.8)	6.0 (6.5)	33.2 (27.0)	25.4 (21.3)	36.8 (32.2)
Baseline FEV_1_, mL	2972 (758)	3631 (744)	3230 (927)	3818 (781)	2123 (652)	2989 (806)	2308 (649)
Baseline FEV_1_/FVC, %	74.1 (7.1)	80.6 (5.8)	78.2 (9.2)	81.6 (6.5)	70.5 (10.5)	75.7 (8.0)	74.7 (7.8)
**Cohort:**	**KORA**	**LBC1921**	**LBC1936**	**PIVUS**	**RS**	**SAPALDIA**	**SHIP**
No. of participants	890	512	1,002	818	1,321	1,401	1,760
No. of FEV_1_ measurements	1,597	706	1,790	1,469	2,016	2,692	2,571
No. of FEV_1_ per person	2	2	2	2	2	2	2
Follow-up duration, yr	3.2	8.9	4.8	5.8	8.3	10.9	7.9
Males, %	47.2	41.4	50.8	49.9	45.1	48	49.4
Baseline age, yr	53.8 (4.5)	79.1 (0.6)	69.6 (0.8)	70.2 (0.2)	74.4 (5.6)	41.1 (11.2)	52.4 (13.6)
Baseline height, cm	169.3 (9.3)	163.2 (9.4)	166.5 (8.9)	169.0 (9.3)	167.3 (9.1)	169.4 (9.1)	169.5 (9.7)
Current smokers, %	20.5	7.0	12.9	10.2	11.1	26.9	32.8
Former smokers, %	40.9	50.4	42.6	39.6	56.7	25.8	23.8
Baseline pack-years†	11.2 (17.1)	15.3 (22.3)	16.9 (25.8)	14.3 (15.8)	25.7 (21.3)	17.4 (18.0)	11.3 (11.9)
Baseline FEV_1_, mL	3280 (792)	1887 (625)	2371 (687)	2452 (682)	2215 (652)	3516 (861)	3238 (876)
Baseline FEV_1_/FVC, %	77.5 (6.2)	79.0 (11.8)	78.3 (10.2)	76.0 (10.0)	74.8 (7.9)	78.5 (8.2)	83.1 (6.6)

*Definition of abbreviations*: ARIC  =  Atherosclerosis Risk in Communities; B58C  =  British 1958 Birth Cohort; BHS  =  Busselton Health Study; CARDIA  =  Coronary Artery Risk Development in Young Adults; CHS  =  Cardiovascular Health Study  =  FHS, Framingham Heart Study; Health ABC  =  Health, Aging, and Body Composition; KORA  =  Cooperative Health Research in the Region of Augsburg; LBC1921  =  Lothian Birth Cohort 1921; LBC1936  =  Lothian Birth Cohort 1936; PIVUS  =  Prospective Investigation of the Vasculature in Uppsala Seniors; RS  =  Rotterdam Study; SAPALDIA  =  Swiss Study on Air Pollution and Lung Diseases in Adults; SD  =  standard deviation; SHIP  =  Study of Health in Pomerania.

^*^Data are presented as mean (SD) unless otherwise indicated; total no. participants  =  27,249, total no. FEV_1_ measurements  =  62,130.

† Pack-years are calculated among current and former smokers at study baseline.

Studies performed genotyping following standard quality control measures; imputation was conducted based on the HapMap CEU reference panel to generate genotype dosages for ∼ 2.5 million autosomal single nucleotide polymorphisms (SNPs) (Table S1 in File S1).

### Statistical analysis

For the analysis of repeated measurement data such as longitudinal change in lung function, mixed effects models offer more flexibility and statistical power than alternative approaches; the model allows for the use of unbalanced data and does not exclude individuals with incomplete records. Each cohort study performed the GWAS using a linear mixed effects model. The model included a random intercept and a random slope, and fixed effects for time (a continuous variable quantifying the time distance between each FEV_1_ measurement and baseline), SNP and its interaction with time (SNP-by-time), baseline age, gender, standing height, smoking pattern during follow-up and its interaction with time (smoking-by-time), baseline smoking pack-years, study site, and principal components for genetic ancestry (as needed). Cohort-specific results for the SNP-by-time interaction term, which estimates the effect of genotype on the rate of change in FEV_1_, were shared, and two meta-analyses, one using all 14 studies and the other using the five studies with ≥3 FEV_1_ measurements per participant, were performed using METAL software with inverse variance weighting to combine effect estimates after applying genomic control correction.[14]

We sought corroborative evidence for SNPs with *P* < 1 × 10^-5^ in the AGES-Reykjavík cohort study (n  =  1,494), and in LHS (n  =  4,048), a clinical cohort study of smokers with mild COPD, in which a longitudinal GWAS was recently reported.[15]

### Gene expression analyses

Expression profiles of genes at the novel loci were evaluated in human lung tissues and primary cell samples using RT-PCR (Table S7 in File S1). Using publicly available data from the Lung Genomics Research Consortium (LGRC), expression profiles of these genes were compared in lung specimens of 219 COPD patients and 137 controls, and sentinel (most associated) SNPs at the novel loci were also searched against an eQTL database of lymphoblastoid cell lines.[16]

This manuscript follows the PRISMA statement and a checklist is available online (Checklist S1).

## Results

### Population characteristics

The majority of the 14 cohort studies had FEV_1_ at two times, but five studies (BHS, CARDIA, CHS, FHS, Health ABC) had ≥3 FEV_1_ measurements per participant. The maximum length of follow-up ranged from 4 to 29 years. Studies with older participants generally had fewer current smokers and more former smokers, and had lower mean baseline FEV_1_.

### Smoking patterns and rate of decline in FEV_1_

All 14 studies implemented a preliminary mixed model adjusted for all specified variables except the SNP terms and reported the estimated rate of change in FEV_1_ by smoking pattern (Table 2). The rate of decline in FEV_1_ in never smokers ranged from 10.0 to 39.7 mL/year, and was generally steeper in studies with older participants, as expected.[4] Across all 14 studies, the meta-analyzed rate of change in FEV_1_ was a decline of 26.9±0.3 mL/year in never smokers, and was 8.8±0.7, 2.6±0.6, and 2.3±0.5 mL/year *steeper* in persistent, intermittent, and former smokers, respectively (Table 2). We repeated the meta-analyses in the five cohort studies with ≥3 FEV_1_ measurements per participant, and found similar, although less statistically significant results.

**Table 2:** Model estimates for the rate of change in FEV_1_ in never smokers and effects of other smoking patterns (compared with never smokers) on the rate of change in FEV_1_ (mL/year)*.

Study	Annual FEV_1_ change in never smokers		Additional Effect† of smoking patterns on annual FEV_1_ change					
	(referent group)		Persistent smokers		Intermittent smokers		Former smokers	
	β	SE	β	SE	β	SE	β	SE
ARIC	−14.0	1.3	−12.4	1.7	−5.5	2.1	−5.3	1.4
B58C	−29.6	1.5	−9.4	2.8	−2.2	3.4	−3.0	3.0
BHS	−23.0	1.0	−20.0	3.0	−8.0	2.0	−9.0	2.0
CARDIA	−26.4	0.5	−6.7	1.3	−0.2	1.0	1.0	1.2
CHS	−35.0	1.1	−2.2	3.3	−4.6	2.2	−2.4	1.7
FHS	−26.0	0.6	−8.1	1.3	−2.9	1.0	−1.1	0.8
Health ABC	−39.7	1.3	−12.9	6.1	−6.8	4.4	−2.6	1.7
KORA	−22.1	3.7	2.2	7.2	−10.4	9.3	2.8	5.2
LBC1921	−10.0	3.6	−11.6	15.7	2.8	14.4	−18.8	4.9
LBC1936	−32.3	3.6	−19.0	9.9	40.1	16.8	4.3	5.3
PIVUS	−21.1	2.5	−15.9	8.2	−21.7	13.4	−3.9	3.9
RS	−27.5	3.7	−1.8	9.0	9.3	8.6	−4.6	4.5
SAPALDIA	−29.7	1.2	−7.4	2.3	−2.0	2.6	−2.8	2.1
SHIP	−31.8	2.8	−0.4	10.9	−0.1	3.9	−15.0	7.3
14-cohort meta-analyzed estimate	−26.9	0.3	−8.8	0.7	−2.6	0.6	−2.3	0.5

*Definition of abbreviations*: ARIC  =  Atherosclerosis Risk in Communities; B58C  =  British 1958 Birth Cohort; BHS  =  Busselton Health Study; CARDIA  =  Coronary Artery Risk Development in Young Adults; CHS  =  Cardiovascular Health Study; FHS  =  Framingham Heart Study; Health ABC  =  Health, Aging, and Body Composition; KORA  =  Cooperative Health Research in the Region of Augsburg; LBC1921  =  Lothian Birth Cohort 1921; LBC1936  =  Lothian Birth Cohort 1936; PIVUS  =  Prospective Investigation of the Vasculature in Uppsala Seniors; RS  =  Rotterdam Study; SAPALDIA  =  Swiss Study on Air Pollution and Lung Diseases in Adults; SE  =  standard error; SHIP  =  Study of Health in Pomerania.

^*^Data shown are the effect estimates (β and SE) of the time and smoking-by-time interaction terms in the preliminary mixed effects model fully adjusted for all specified variables except the SNP terms. Time represents the rate of change in FEV_1_ in never smokers and the smoking-by-time interaction term represents the effects of the other three smoking patterns on the rate of change in FEV_1_, compared with never smokers. Smoking categories are defined as persistent (smoke throughout follow-up), intermittent (stop and/or start smoking during follow-up) and former (smoke only prior to start of follow-up).

† Effect estimates in smoking categories are added to estimates in never smokers to compute the actual rate of change in each group (for example, in ARIC, the point estimate of the rate of change in FEV_1_ in persistent smokers was −14.0 − 12.4  =  −26.4 mL/year).

### Discovery meta-analyses

Study-specific genomic inflation factors (λ_gc_) were calculated for the SNP-by-time interaction term and used for study-level genomic control prior to the meta-analyses. Study-specific λ_gc_ values ranged from 0.96 to 1.11 (Table S1 in File S1) and the meta-analysis λ_gc_ was 1.01 for both the 14-study and five-study meta-analyses. Figures S1 and S2 in File S1 present the Manhattan and quantile-quantile (QQ) plots.

**Figure 1:**
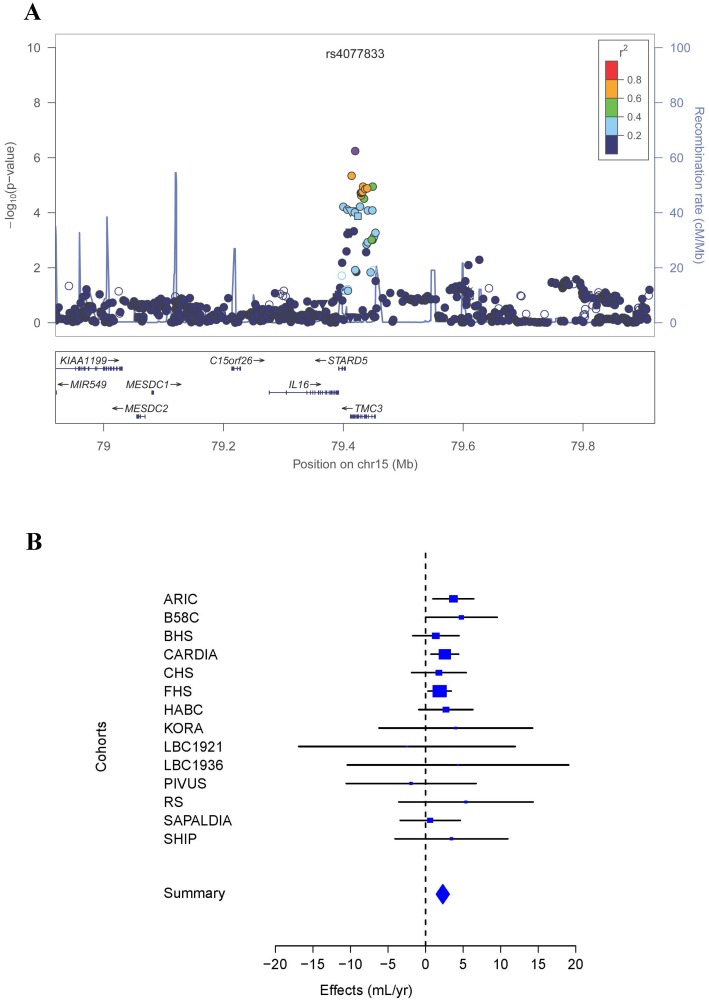
Association of the chromosome 15 locus with the rate of change in FEV_1_ in the meta-analysis of 14 cohort studies. **A**) Regional association plot, where the X-axis is Megabase (Mb) position and Y-axes are the negative log of the *P* value on the left and recombination rate on the right. The sentinel SNP is colored in purple and linkage disequilibrium to the sentinel SNP is depicted by degree of color according to the legend. **B**) Forest plot for rs4077833, where the size of the square for each study represents its contributing weight to the meta-analysis.

In the meta-analysis including all 14 cohort studies, 15 SNPs at nine independent loci were associated with the rate of change in FEV_1_ at *P* < 1 × 10^−5^, and none reached the genome-wide significance threshold of *P* < 5 × 10^−8^. The association results for the sentinel SNPs at these nine loci are presented in Table 3, and more detailed results for all 15 SNPs are included in Table S2 in File S1. The most statistically significant association, and the only one that reached *P* < 1 × 10^−6^, was for rs4077833, an intronic SNP located in the novel *IL16/STARD5/TMC3* gene region on chromosome 15 (*P*  =  5.71 × 10^−7^; Figures 1A and 1B). The C allele of rs4077833, with a frequency of 10%, was associated with an attenuation of the rate of decline in FEV_1_ by 2.3 mL/year in comparison to the G allele.

**Table 3:** Association of the most statistically significant SNPs with the rate of change in FEV_1_ (mL/year) in the meta-analysis of 14 cohort studies (n  =  27,249)*.

SNP	Chr	Position	Closest Gene(s)	Coded Allele	Frequency	β	SE	*P* Value
rs12137475	1	44059735	*ST3GAL3*	T	0.11	−3.5	0.8	3.90 × 10^−6^
rs766488	1	61583103	*NFIA*	A	0.31	1.4	0.3	6.60 × 10^−6^
rs17698444	1	215483178	*ESRRG/GPATCH2*	C	0.89	−2.2	0.5	2.62 × 10^−6^
rs12692550	2	159958017	*BAZ2B*	T	0.17	−1.7	0.4	5.16 × 10^−6^
rs2260722	13	113236292	*TMCO3*	A	0.72	−1.5	0.3	1.83 × 10^−6^
rs4077833	15	79419738	*IL16/STARD5/TMC3*	C	0.10	2.3	0.5	5.71 × 10^−7^
rs8027498	15	89595638	*SV2B*	A	0.25	1.4	0.3	9.41 × 10^−6^
rs8051319	16	15794449	*MYH11*	T	0.60	1.7	0.3	5.12 × 10^−6^
rs740557	17	62451139	*CACNG4*	C	0.85	−2.3	0.5	3.59 × 10^−6^

*Definition of abbreviations*: Chr  =  chromosome; SE  =  standard error; SNP  =  single-nucleotide polymorphism.

^*^Data reported are the meta-analysis results of the SNP-by-time interaction term from the GWAS mixed effects model. A positive β-coefficient indicates an attenuation of FEV_1_ decline and a negative β-coefficient an acceleration of FEV_1_ decline.

**Figure 2:**
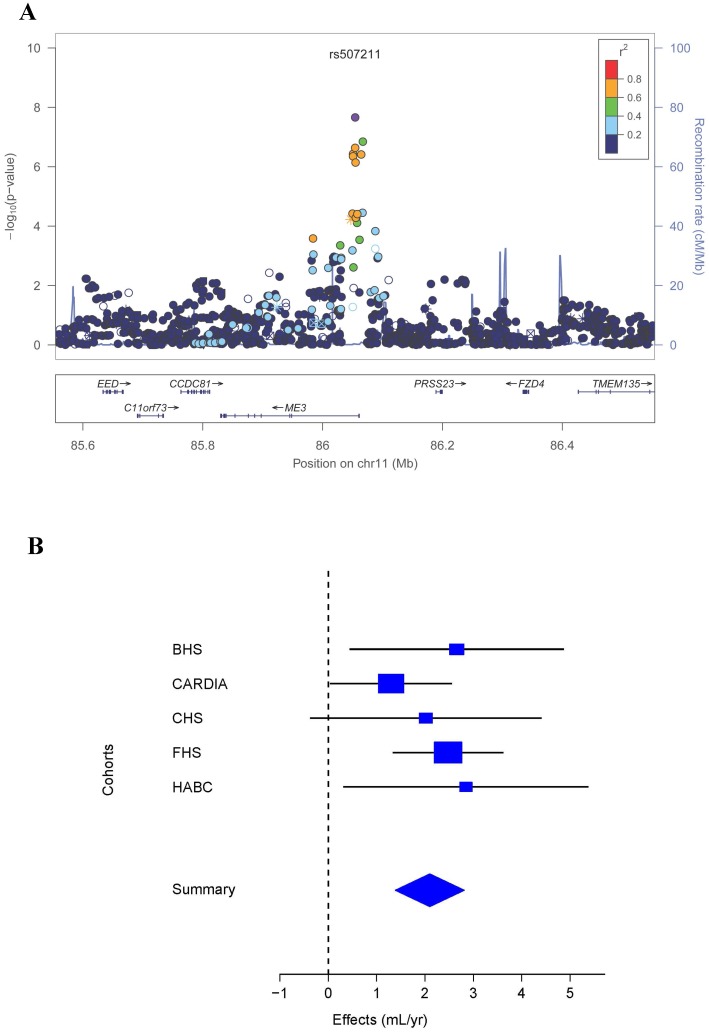
Association of the chromosome 11 locus with the rate of change in FEV_1_ in the meta-analysis of the five cohort studies with ≥3 FEV_1_ measurements per participant. **A**) Regional association plot, where the X-axis is Megabase (Mb) position, and the Y-axes are the negative log of the *P* value on the left and recombination rate on the right. The sentinel SNP is colored in purple and linkage disequilibrium to the sentinel SNP is depicted by degree of color according to the legend. **B**) Forest plot for rs507211, where the size of the square for each study represents its contributing weight to the meta-analysis.

For estimation of longitudinal trajectory in lung function, having more than two measurements over time provides greater precision.[4] We performed a further meta-analysis with the five cohort studies (BHS, CHS, CARDIA, FHS, Health ABC) having ≥3 FEV_1_ measurements per participant, with a combined sample size of 10,476 participants and 32,054 FEV_1_ measurements (Methods S1 in File S1 for further details). A novel region on chromosome 11 had a genome-wide significant association (*P* < 5 × 10^−8^) with the rate of change in FEV_1_ (Table 4). The most statistically significant finding at this locus was for rs507211, an intronic SNP located in *ME3* (Figures 2A and 2B). Six other SNPs, which are in linkage disequilibrium (LD) with rs507211 and are located in *ME3*, were identified at *P* < 1 × 10^−6^ (Table S3 in File S1). The rs507211 A allele, with a frequency of 25%, was associated with an attenuation of the rate of decline in FEV_1_ by 2.09 mL/year in comparison to the G allele (*P*  =  2.18 × 10^−8^). Besides the *ME3* locus, 17 SNPs from four other chromosomal regions had *P* values between 5 × 10^−8^ and 1 × 10^−5^ for associations with the rate of change in FEV_1_ (Tables 4 and Table S3 in File S1).

**Table 4:** Association of the most statistically significant SNPs with the rate of change in FEV_1_ (mL/year) in the meta-analysis of the five cohort studies with ≥3 FEV_1_ measurements per participant (n  =  10,476).

SNP	Chr	Position	Closest Gene(s)	Coded Allele	Frequency	β*	SE	*P* Value
rs10209501	2	28536881	*FOSL2/PLB1*	A	0.33	1.6	0.4	7.09 × 10^−6^
rs12692550	2	159958017	*BAZ2B*	T	0.18	−2.0	0.4	2.02 × 10^−6^
rs1729588	3	110790025	*FLJ25363/MIR4445*	A	0.30	1.6	0.4	8.38 × 10^−6^
rs10764053	10	19863644	*C10orf112*	T	0.47	1.5	0.3	4.15 × 10^−6^
rs507211	11	86054387	*ME3*	A	0.25	2.1	0.4	2.18 × 10^−8^

*Definition of abbreviations*: Chr  =  chromosome; SE  =  standard error; SNP  =  single-nucleotide polymorphism.

^*^Data reported are the meta-analysis results of the SNP-by-time interaction term from the GWAS mixed effects model. A positive β-coefficient indicates an attenuation of FEV_1_ decline and a negative β-coefficient an acceleration of FEV_1_ decline.

### Additional analyses

Corroborative evidence was sought for the sentinel SNP at each of the 14 loci associated at *P* < 1 × 10^−5^ (from both the 14-study and five-study meta-analyses) in 1,494 adults from the AGES-Reykjavík population-based cohort study (Table S4 in File S1). A *P* value of 0.004, representing the Bonferroni correction for 14 tests at the α  =  0.05 level, was selected *a priori* as the threshold for statistical significance. No SNPs achieved this threshold. The lowest *P* value was for rs740577 in *CACNG4* (*P*  =  0.08), which showed consistent effect direction and magnitude with the original meta-analysis.

These same 14 SNPs were further examined in LHS, a clinical cohort study of 4,048 smokers with mild COPD for evidence of consistent association between healthy and diseased individuals.[17] None of the 14 SNPs were associated with the rate of change in FEV_1_ in LHS at *P* < 0.004 (Table S4 in File S1).

Previous meta-analyses in the CHARGE and SpiroMeta consortia identified 26 novel loci associated with cross-sectional FEV_1_ and/or FEV_1_/FVC at genome-wide significance.[9]-[11] We examined the sentinel SNPs from these loci in the meta-analysis of the 14 cohort studies for association with the rate of change in FEV_1_ (Table S5 in File S1). Given the *a priori* association with cross-sectional lung function, a *P* value threshold of 0.05 was used. Sentinel SNPs in *PID1*, *HHIP*, *GPR126*, and *CFDP1* showed association with the rate of change in FEV_1_ (0.005 ≤ *P* ≤ 0.048).

### Gene expression analyses

Three genes (*IL16*, *STARD5*, and *TMC3*) at the novel chromosome 15 locus and *ME3* at the novel chromosome 11 locus were selected for follow-up mRNA expression profiling in human lung tissue, and primary cultures of human bronchial epithelial and airway smooth muscle cells, together with control tissues (peripheral blood mononuclear cells and brain). Transcripts of *STARD5* and *ME3* were found in all lung-derived tissues, transcripts of *IL16* were found in lung tissue and smooth muscle cells, but not in epithelial cells, and *TMC3* was not expressed in any of the lung-derived tissues (Table S6 in File S1).

Using the public LGRC data repository, we found that the expression profiles of *IL16*, *STARD5*, and *ME3* in human lung samples showed statistically significant differences (*P* < 0.05) between COPD patients and controls (Figure S3 in File S1). Lower levels of *IL16* (*P*  =  0.004) were observed in COPD patients compared with controls, whereas higher levels of *STARD5* (*P*  =  3.22 × 10^-9^) and *ME3* (*P*  =  0.044) were observed in COPD patients compared with controls. Data on *TMC3* expression were not available.

We performed additional follow-up analysis of the sentinel SNPs at the two novel loci using an eQTL database of lymphoblastoid cell lines (Table S8 in File S1). Trans-eQTL associations were observed between rs4077833 at the *IL16/STARD5/TMC3* locus and a nuclear receptor, *NR1I2* (chromosome 3; *P*  =  6.84 × 10^-4^) and between rs507211 at the *ME3* locus and *KIAA1109* (chromosome 4; *P*  =  5.20 × 10^-4^), which is part of a gene cluster (*KIAA1109-TENR-IL2-IL21*) that encodes two interleukins (IL2 and IL21).[18]

## Discussion

Although the genetic contribution to cross-sectional lung function phenotypes has been addressed by large-scale GWAS, much less information is available for longitudinal lung function phenotypes. To identify novel loci that specifically affect lung function change over time, we performed a large-scale GWAS of the rate of change in FEV_1_ in 27,249 participants from 14 population-based cohort studies. We identified a novel locus (*IL16/STARD5/TMC3*) on chromosome 15 with suggestive evidence for association with the rate of change in FEV_1_. Given the greater precision to estimate longitudinal trends with more measurements, a meta-analysis of the five cohort studies with ≥3 FEV_1_ measurements per participant was performed, and it identified a second novel locus (*ME3*) on chromosome 11 at genome-wide statistical significance. For both loci, the minor allele was protective, and the magnitude of the association with the rate of change in FEV_1_ was similar to that of being an intermittent or former smoker versus a never-smoker.

The sentinel SNP at the novel chromosome 15 locus is located in *TMC3*, although two neighboring genes, *IL16* and *STARD5* both harbor SNPs that are in modest LD with the sentinel SNP (Figure 1A). TMC3, a member of the transmembrane channel-like gene family, likely functions as an ion channel, transporter, or modifier,[19] and has been associated with deafness and skin cancer.[20], [21] IL16 is a pleiotropic immunomodulatory cytokine that acts as a chemoattractant for CD4^+^ cells and contributes to their recruitment and activation in response to inflammation.[22] Notably, asthma was the first disease where increased *IL16* expression was observed.[23] Subsequent studies confirmed that in the non-diseased state *IL16* is almost exclusively expressed by T lymphocytes in lymphatic tissue, whereas in asthmatic patients IL16 is also synthesized by airway epithelial cells to inhibit airway inflammation.[24]-[26] A promoter polymorphism (T-295C) in *IL16* was associated with asthma in a Caucasian population in England,[27] although this finding was not confirmed in an Australian study.[28] STARD5 belongs to the steroidogenic acute regulatory lipid transfer domain protein superfamily, and is involved in the trafficking of cholesterol and other lipids between intracellular membranes.[29] Recent *in vitro* studies showed increased *STARD5* expression and protein redistribution as a protective mechanism in response to induced endoplasmic reticulum (ER) stress and consequent over-accumulation of intracellular free cholesterol.[30] We confirmed the expression of *STARD5* in all human lung tissues examined and of *IL16* in human lung smooth muscle cells, but not epithelial cells, in line with previous observations. In contrast, no expression of *TMC3* was detected in any of the tested human lung tissues. We also found significantly lower levels of *IL16* in whole lung samples from COPD patients compared with controls, in contrast to its increased expression in asthma, and significantly higher levels of *STARD5* in COPD patients compared with controls. Taken together, these results suggest *IL16* as the most likely candidate accounting for the observed association, but further investigation is needed to elucidate underlying mechanisms.

The sentinel SNP at the novel chromosome 11 locus is located in *ME3*, whose protein product is a mitochondrial NADP(+)-dependent malic enzyme that catalyzes the oxidative decarboxylation of malate to pyruvate using NADP+ as a cofactor.[31] Mitochondrial malic enzymes play a role in the energy metabolism in tumors, and are considered potential therapeutic targets in cancer.[32], [33] We performed independent expression profiling of *ME3* and confirmed its expression in all human lung tissues examined, and found significantly higher levels of *ME3* in lung samples from COPD patients compared with controls. In addition, we looked up the sentinel SNP in *ME3* in a recent GWAS of airway obstruction and found a *P* value of 0.049.[34] Taken together, these results support *ME3* as a biologically plausible candidate in the regulation of lung function and pathogenesis of COPD.

The identification of trans-eQTL associations for the sentinel SNPs at both the *IL16/STARD5/TMC3* and *ME3* loci is interesting, and while the interpretation of trans-eQTL associations is ambiguous,[35] the regions these SNPs regulate merit further study.

Besides the GWAS meta-analyses, the assembly of 14 longitudinal cohort studies allowed us to meta-analyze the association of cumulative smoking patterns with the rate of change in FEV_1_ in the general population. The meta-analyzed estimate for the rate of decline in FEV_1_ in never smokers was 26.9 mL/year, and the annual decline was steeper in persistent, intermittent, and former smokers by 8.8, 2.6, and 2.3 mL/year, respectively. These findings provide a reference point for the effect of cigarette smoking on longitudinal lung function change in the general population.

There is phenotypic variation among the 14 cohort studies in aspects such as baseline age and cigarette smoking, and in factors that are of special importance to this longitudinal GWAS, such as the number of FEV_1_ measurements per participant and follow-up duration. Phenotypic heterogeneity represents a general challenge in genetic epidemiology, particularly in the investigation of longitudinal phenotypes. Thus, we performed a meta-analysis using the subset of cohort studies with ≥3 FEV_1_ measurements per participant, given that longitudinal trajectories are best estimated over longer time periods and with more measurements. There was little overlap between the top loci identified in the two meta-analyses at *P* < 1 × 10^−5^, suggesting that phenotypic heterogeneity affected the association results. Future meta-studies of lung function decline should aim to increase sample size while maintaining high phenotypic comparability among participating studies. In addition, the trajectory of lung function change, especially over a long period of time, is known to be nonlinear, which may require the use of nonlinear time effects in the statistical model. In this study, given that over half of the included cohort studies have FEV_1_ measurements at only two time points, our consideration was limited to a linear time effect. Further, the outcome studied, the rate of change in lung function, represents one of many ways to describe lung function change. Additional studies of other aspects of lung function change, such as reduced growth and premature decline, would be of interest.

We sought corroborative evidence in a single cohort study of 1,494 participants. This sample size is much smaller and arguably insufficient compared with replications applied to previous studies of cross-sectional lung function phenotypes. Thus, despite the lack of corroboration for the two novel loci identified in the meta-analyses, results from the complementary gene expression analyses provide compelling evidence for biologically plausible roles of the implicated genes in the longitudinal change in lung function.

None of the 14 sentinel SNPs were associated with the rate of change in FEV_1_ in the COPD patient-based LHS cohort. Similarly, a previous population-based GWAS of lung function decline noted a high degree of heterogeneity in findings when analyses were stratified by presence/absence of asthma.[12] The observed discrepancy of association results suggests that the genetic determination of lung function decline may be different in healthy individuals compared with COPD patients, may contribute differentially in a pre-diseased vs. post-diseased state in which medications may influence the rates of decline, or that LHS was underpowered for confirming our findings.

In this study, statistical models included a comprehensive list of confounders that are commonly adjusted for when modeling lung function phenotypes. Given the study's meta-analysis design and the objective to carry out the same statistical model in all cohort studies, additional covariates that were not available in all cohort studies could not be included. In addition, the adjustment of certain confounders, such as smoking, is challenging in a longitudinal study, and although we accounted for the two most important aspects of smoking, cumulative pattern and dosage, residual confounding due to smoking cannot be excluded.

In summary, we performed GWAS of the longitudinal change in lung function and subsequent meta-analyses, using harmonized data from more than 27,000 participants of European ancestry to identify genetic loci influencing the rate of change in FEV_1_. We identified the novel *ME3* locus on chromosome 11 at genome-wide significance and found suggestive evidence for association at the novel *IL16/STARD5/TMC3* locus on chromosome 15. Additional expression analyses confirmed the expression of *ME3*, *IL16*, and *STARD5* in multiple lung tissues, and found differential expression profiles of these three genes in the lungs of COPD patients compared to non-COPD controls. These results support the involvement of these implicated genes in the longitudinal change in lung function in adults of European ancestry. Additional studies with larger sample size and in populations of other races/ethnicities are warranted.

## Supporting Information

File S1 **This is a single file that contains all supporting information for the paper.** Briefly, File S1 contains the following items: Methods S1, which describes further details of the cohort studies and the statistical methodology; Table S1, Details of SNP genotyping, quality control (QC), imputation, and statistical analysis across the 14 cohort studies; Table S2, Regression results for single nucleotide polymorphisms associated with the rate of change in FEV_1_ (mL/year) at *P* < 1 × 10^-5^ in the meta-analysis of 14 cohort studies (N = 27,249); Table S3, Regression results for single nucleotide polymorphisms associated with the rate of change in FEV_1_ (mL/year) at *P* < 1 × 10^-5^ in the meta-analysis of the five cohort studies with three or more FEV_1_ measurements per participant (N = 10,476); Table S4, Association of the 14 sentinel SNPs from the meta-analyses in the AGES-Reykjavík study (AGES) and the Lung Health Study (LHS) for the rate of change in FEV_1_ (mL/year); Table S5, Association of previously reported loci in GWAS of cross-sectional lung function with the rate of change in FEV_1_ (mL/year) in the meta-analysis of 14 cohort studies (N = 27,249); Table S6, mRNA expression profiling of the implicated genes at the two novel loci in human lung and control tissues; Table S7, Primers for mRNA expression profiling; Table S8, Summary of eQTL look-up for the most significant SNPs at the novel chromosome 11 and 15 loci; Figure S1, Manhattan and QQ plots for the meta-analysis of the rate of change in FEV_1_ in 14 cohort studies; Figure S2, Manhattan and QQ plots for the meta-analysis of the rate of change in FEV_1_ in the five cohort studies with three or more FEV_1_ measurements per participant; Figure S3, mRNA expression profiling in human lung samples from 219 COPD patients and 137 controls for A) *IL16*, B) *STARD5*, and C) *ME3*, using publicly available microarray data from the Lung Genomics Research Consortium site (http://www.lung-genomics.org/). The y-axes reflect the probe intensities of each gene transcript in the binary logarithm form, with the red dots indicating the average probe intensities and the red bars indicating standard deviation. The *P* values were calculated using the two-sample t-test.
(DOCX)

Checklist S1 **PRISMA Checklist.**
(DOCX)
