# Supplementary material for: Large-Scale Genome-Wide Association Studies and Meta-Analyses of Longitudinal Change in Adult Lung Function
Source: PLoS One. 2014 Jul 1;9(7):e100776. doi: 10.1371/journal.pone.0100776 (PMC4077649; doi:10.1371/journal.pone.0100776)
Supplement: File S1 — This is a single file that contains all supporting information for the paper. Briefly, File S1 contains the following items: Methods S1, which describes further details of the cohort studies and the statistical methodology; Table S1, Details of SNP genotyping, quality control (QC), imputation, and statistical analysis across the 14 cohort studies; Table S2, Regression results for single nucleotide polymorphisms associated with the rate of change in FEV1 (mL/year) at P < 1 × 10^-5^ in the meta-analysis of 14 cohort studies (N = 27,249); Table S3, Regression results for single nucleotide polymorphisms associated with the rate of change in FEV1 (mL/year) at P < 1 × 10^-5^ in the meta-analysis of the five cohort studies with three or more FEV1 measurements per participant (N = 10,476); Table S4, Association of the 14 sentinel SNPs from the meta-analyses in the AGES-Reykjavík study (AGES) and the Lung Health Study (LHS) for the rate of change in FEV1 (mL/year); Table S5, Association of previously reported loci in GWAS of cross-sectional lung function with the rate of change in FEV1 (mL/year) in the meta-analysis of 14 cohort studies (N = 27,249); Table S6, mRNA expression profiling of the implicated genes at the two novel loci in human lung and control tissues; Table S7, Primers for mRNA expression profiling; Table S8, Summary of eQTL look-up for the most significant SNPs at the novel chromosome 11 and 15 loci; Figure S1, Manhattan and QQ plots for the meta-analysis of the rate of change in FEV1 in 14 cohort studies; Figure S2, Manhattan and QQ plots for the meta-analysis of the rate of change in FEV1 in the five cohort studies with three or more FEV1 measurements per participant; Figure S3, mRNA expression profiling in human lung samples from 219 COPD patients and 137 controls for A) IL16, B) STARD5, and C) ME3, using publicly available microarray data from the Lung Genomics Research Consortium site (http://www.lung-genomics.org/). The y-axes reflect the probe intensities o [file pone.0100776.s001.docx]

**File S1 Supporting Information for:**

**Large-scale Genome-Wide Association Studies and Meta-analyses of Longitudinal Change in Adult Lung Function**

Wenbo Tang^1^*, Matthew Kowgier^2^*, Daan W. Loth^3,4^*, María Soler Artigas^5,6^*, Bonnie R. Joubert^7^*, Emily Hodge^8^*, Sina A. Gharib^9^*, Albert V. Smith^10,11^, Ingo Ruczinski^12^, Vilmundur Gudnason^10,11^, Rasika A. Mathias^13^, Tamara B. Harris^14^, Nadia N. Hansel^13^, Lenore J. Launer^14^, Kathleen C. Barnes^13^, Joyanna G. Hansen^1^, Eva Albrecht^15^, Melinda C. Aldrich^16^, Michael Allerhand^17^, R. Graham Barr^18,19^, Guy G. Brusselle^3,20,21^, David J. Couper^22^, Ivan Curjuric^23,24^, Gail Davies^17,25,26^, Ian J. Deary^17,26^, Josée Dupuis^27,28^, Tove Fall^29^, Millennia Foy^30^, Nora Franceschini^31^, Wei Gao^27^, Sven Gläser^32^, Xiangjun Gu^30^, Dana B. Hancock^7,33^, Joachim Heinrich^34^, Albert Hofman^3,35^, Medea Imboden^23,24^, Erik Ingelsson^29,36^, Alan James^37^, Stefan Karrasch^38,39,40^, Beate Koch^32^, Stephen B. Kritchevsky^41^, Ashish Kumar^23,24,36^, Lies Lahousse^3,20^, Guo Li^42^, Lars Lind^43^, Cecilia Lindgren^36,44^, Yongmei Liu^45^, Kurt Lohman^46^, Thomas Lumley^47^, Wendy L. McArdle^48^, Bernd Meibohm^49^, Andrew P. Morris^36^, Alanna C. Morrison^50^, Bill Musk^37^, Kari E. North^31^, Lyle J. Palmer^2,51,52^, Nicole M. Probst-Hensch^23,24^, Bruce M. Psaty^42,53,54,55^, Fernando Rivadeneira^35,56^, Jerome I. Rotter^57^, Holger Schulz^34^, Lewis J. Smith^58^, Akshay Sood^59^, John M. Starr^17,60^, David P. Strachan^61^, Alexander Teumer^62^, André G. Uitterlinden^35,56^, Henry Völzke^63^, Arend Voorman^64^, Louise V. Wain^6,65^, Martin T. Wells^66^, Jemma B. Wilk^28,67^, O. Dale Williams^68^, Susan R. Heckbert^42,53,54^, Bruno H. Stricker^3,4^, Stephanie J. London^7^, Myriam Fornage^30,50^**, Martin D. Tobin^5,6^**, George T. O’Connor^28,69^**, Ian P. Hall^8^**, Patricia A. Cassano^1,70^**

*starred first authors

**starred last authors

**Affiliations:**

1. Division of Nutritional Sciences, Cornell University, Ithaca, NY, USA
2. Ontario Institute for Cancer Research, Toronto, Ontario, Canada and Biostatistics Division, Dalla Lana School of Public Health, University of Toronto
3. Department of Epidemiology, Erasmus Medical Center, Rotterdam, the Netherlands
4. Netherlands Healthcare Inspectorate, The Hague, the Netherlands
5. University of Leicester, Genetic Epidemiology Group, Department of Health Sciences, Leicester, UK
6. National Institute for Health Research (NIHR) Leicester Respiratory Biomedical Research Unit, Glenfield Hospital, Leicester, UK
7. Epidemiology Branch, National Institute of Environmental Health Sciences, National Institutes of Health, U.S. Department of Health and Human Services, Research Triangle Park, NC, USA
8. Division of Respiratory Medicine, University Hospital of Nottingham, Nottingham, UK
9. Computational Medicine Core, Center for Lung Biology, Division of Pulmonary & Critical Care Medicine, Department of Medicine, University of Washington, Seattle, WA, USA
10. Icelandic Heart Association, Kopavogur, Iceland
11. University of Iceland, Reykjavik, Iceland
12. Department of Biostatistics, Bloomberg School of Public Health, Johns Hopkins University, Baltimore, MD, USA
13. Department of Medicine, School of Medicine, Johns Hopkins University, Baltimore, MD, USA
14. Laboratory of Epidemiology, Demography, and Biometry, National Institute on Aging, National Institutes of Health, Bethesda, MD, USA
15. Institute of Genetic Epidemiology, Helmholtz Zentrum München - German Research Center for Environmental Health, Neuherberg, Germany
16. Department of Thoracic Surgery and Division of Epidemiology, Vanderbilt University Medical Center, Nashville, TN, USA
17. Centre for Cognitive Ageing and Cognitive Epidemiology, University of Edinburgh, Edinburgh, UK
18. Division of General Medicine, Pulmonary, Allergy and Critical Care, Department of Medicine, College of Physicians and Surgeons, Columbia University, New York, NY, USA
19. Department of Epidemiology, Mailman School of Public Health, Columbia University, New York, NY, USA
20. Department of Respiratory Disease, University Hospital Ghent, Ghent, Belgium
21. Department of Respiratory Medicine, Erasmus Medical Center, Rotterdam, the Netherlands
22. Department of Biostatistics, University of North Carolina at Chapel Hill, Chapel Hill, NC, USA
23. Swiss Tropical and Public Health Institute, Basel, Switzerland
24. University of Basel, Basel, Switzerland
25. Medical Genetics Section, University of Edinburgh Molecular Medicine Centre and MRC Institute of Genetics and Molecular Medicine, Western General Hospital, Edinburgh, UK
26. Department of Psychology, University of Edinburgh, Edinburgh, UK
27. Biostatistics Department, Boston University School of Public Health, Boston, MA, USA
28. The National Heart, Lung, and Blood Institute’s Framingham Heart Study, Framingham, MA, USA
29. Department of Medical Sciences, Molecular Epidemiology and Science for Life Laboratory, Uppsala University, Uppsala, Sweden
30. Institute of Molecular Medicine, University of Texas Health Science Center at Houston, Houston, TX, USA
31. Gillings School of Global Public Health, Department of Epidemiology, University of North Carolina at Chapel Hill, Chapel Hill, NC, USA
32. Department of Internal Medicine B; Pneumology, Cardiology, Intensive Care Medicine; Field of Research: Pneumology and Pneumological Epidemiology, University Medicine Greifswald, Germany
33. Behavioral Health Epidemiology Program, Research Triangle Institute, Research Triangle Park, NC, USA
34. Institute of Epidemiology I, Helmholtz Zentrum München - German Research Center for Environmental Health, Neuherberg, Germany and Comprehensive Pneumology Center Munich (CPC-M), Member of the German Center for Lung Research, Munich, Germany
35. Netherlands Consortium for Healthy Aging, Rotterdam, the Netherlands
36. Wellcome Trust Centre for Human Genetics, University of Oxford, Oxford, UK
37. School of Medicine and Pharmacology, University of Western Australia, Perth, Western Australia, Australia
38. Institute and Outpatient Clinic for Occupational, Social and Environmental Medicine, Ludwig-Maximilians-Universität, Munich, Germany
39. Institute of General Practice, University Hospital Klinikum rechts der Isar, Technische Universität München, Munich, Germany
40. Institute of Epidemiology I, Helmholtz Zentrum München - German Research Center for Environmental Health, Neuherberg, Germany
41. Sticht Center on Aging, Wake Forest School of Medicine, Winston-Salem, NC, USA
42. Cardiovascular Health Research Unit, University of Washington, Seattle, WA, USA
43. Department of Medical Sciences, Uppsala University, Uppsala, Sweden
44. Broad Institute of MIT and Harvard, Cambridge, Massachusetts, USA
45. Department of Epidemiology and Prevention, Division of Public Health Sciences, Wake Forest School of Medicine, Winston-Salem, NC, USA
46. Department of Biostatistical Sciences, Division of Public Health Sciences, Wake Forest School of Medicine, Winston-Salem, NC, USA
47. Department of Statistics, University of Auckland, Auckland, New Zealand
48. School of Social and Community Medicine, University of Bristol, Bristol, UK
49. College of Pharmacy, University of Tennessee Health Science Center, Memphis, TN, USA
50. Human Genetics Center, School of Public Health, University of Texas Health Science Center at Houston, Houston, TX, USA
51. Epidemiology and Obstetrics & Gynaecology, University of Toronto, Toronto, Ontario, Canada
52. Samuel Lunenfeld Research Institute, Toronto, Ontario, Canada
53. Department of Epidemiology, University of Washington, Seattle, WA, USA
54. Group Health Research Institute, Group Health Cooperative, Seattle, WA, USA
55. Department of Medicine, University of Washington, Seattle, WA, USA
56. Department of Internal Medicine, Erasmus Medical Center, Rotterdam, the Netherlands
57. Institute for Translational Genomics and Population Sciences, Los Angeles Biomedical Research Institute and Department of Pediatrics at Harbor-UCLA Medical Center, Torrance, CA, USA
58. Northwestern University Feinberg School of Medicine, Chicago, IL, USA
59. University of New Mexico, NM, USA
60. Alzheimer Scotland Dementia Research Centre, University of Edinburgh, Edinburgh, UK
61. Division of Population Health Sciences and Education, St George's, University of London, London, UK
62. Department for Genetics and Functional Genomics, Interfaculty Institute for Genetics and Functional Genomics, University Medicine Greifswald, Greifswald, Germany
63. Institute for Community Medicine, Study of Health In Pomerania (SHIP)/Clinical Epidemiological Research, University Medicine Greifswald, Greifswald, Germany
64. Department of Biostatistics, University of Washington, Seattle, WA, USA
65. Department of Statistical Science, Cornell University, Ithaca, NY, USA
66. Division of Aging, Department of Medicine, Brigham and Women’s Hospital and Harvard Medical School, Boston, MA, USA
67. Florida International University, Miami, FL, USA
68. Section of Pulmonary, Allergy, and Critical Care Medicine, Department of Medicine, Boston University School of Medicine, Boston, MA, USA
69. Department of Health Policy and Research, Division of Biostatistics and Epidemiology, Weill Cornell Medical College, New York, NY, USA

**Methods S1**

Study population

All 14 cohort studies are members of the CHARGE or SpiroMeta Consortium. The respective local Institutional Review Boards approved all study protocols, and written informed consent for genetic studies was obtained from all participants.

Pulmonary function assessment

Study-specific information on spirometry protocols was reported previously ([1](#_ENREF_1)). Additional descriptions of spirometry tests specific to this longitudinal study are provided below.

**ARIC** measurements were completed at two time points (baseline and approximately 3 years later) in accordance with the standardized guidelines of the American Thoracic Society (ATS), as previously described ([2](#_ENREF_2)). Measurements were made with a Collins Survey II water-seal spirometer (Collins Medical, Inc.) and Pulmo-Screen II software (PDS Healthcare Products, Inc.).

The **British 1958 birth cohort (B58C)** is a longitudinal study of all people born in England, Scotland and Wales during one week in 1958. At age 35 years, spirometry was performed in 1156 cohort members with a history of asthma, wheezy bronchitis or pneumonia during childhood or early adult life, and a subsample of 293 cohort members with no history of any of these conditions ([3](#_ENREF_3)). At age 45 years, measurements of ventilatory function were repeated as part of a more general biomedical examination of the entire cohort ([4](#_ENREF_4)). The results contributed to the present meta-analysis relate to 827 individuals with valid spirometry on both occasions ([5](#_ENREF_5)), plus genome-wide genotyping performed on DNA samples collected at the 45-year follow-up. On both occasions, spirometry was performed in the home by trained research nurses, after daily calibration of the instrument using a 1L syringe. Measurements were taken in the standing position, without noseclips. At age 35 years, at least three (and up to eight) forced expiratory maneuvers were recorded by dry bellows spirometer (Vitalograph R-model or S-model; Vitalograph, Buckingham, UK) until two technically satisfactory measurements of forced expiratory volume in the first second (FEV_1_) within 5% of each other were obtained. At age 45 years, at least three (and up to five) spirograms were recorded by pneumotachograph (Micro; Vitalograph, Buckingham, UK) until three technically satisfactory blows had been obtained. On each occasion, the highest technically satisfactory values of FEV_1_ and forced vital capacity (FVC) were used in the analysis.

The **Busselton Health Study (BHS)** is a longitudinal survey of the town of Busselton in the south-western region of Western Australia that began in 1966. Lung function was measured at 9 time points. Spirometric measures of forced expired volume in one second (FEV_1_) and forced vital capacity (FVC) were assessed as described previously ([6](#_ENREF_6), [7](#_ENREF_7)).

Spirometry was performed at **CARDIA** years 0, 2, 5, 10 and 20 examination visits, adhering to the ATS guidelines. At CARDIA years 0, 2, 5 and 10 examination visits, spirometry was performed using the Collins Survey 8-liter water-sealed spirometer and the Eagle II microprocessor (Warren E. Collins, Inc., Braintree, MA) in a sitting position with noseclips, as per the 1979 ATS criteria ([8](#_ENREF_8)). Specifically, each subject performed a minimum of three trials with expirations recorded to the FVC plateau, which occurs after six seconds of expiration and was maintained for at least one second before terminating the forced expiratory maneuver. If, at the end of the three trials, there were at least three acceptable tracings, and with the maximum FVC and FEV_1_ reproduced to within 5% or 100 mL, whichever is greater, no more trials were performed. At CARDIA year 20 examination visit, a dry rolling-seal SensorMedics model 1022 spirometer fitted by OMI (Viasys Corp, Loma Linda, CA) was used for spirometry testing in a standing position with noseclips. The criteria for reproducibility were changed at the year 20 visit - the two largest FVC values were to agree within 150 ml, and the two largest FEV_1_ values were also to agree within 150 ml, consistent with the 1994 update by the ATS ([9](#_ENREF_9)). A comparability study performed on 25 volunteers at the LDS Hospital (Salt Lake City, UT) demonstrated excellent consistency between the old and new machines; the average difference between the Collins Survey and OMI spirometer was 6 mL for FVC and 21 mL for FEV_1_.

In **CHS**, spirometry was completed on three occasions (baseline and after 4 and 7 years of follow-up) for the original cohort recruited in 1989-90. The spirometry procedures for pulmonary function testing have been previously described ([10](#_ENREF_10), [11](#_ENREF_11)). Briefly, spirometry technicians were centrally trained and certified prior to recruitment of participants. A standard spirometry system, including a Collins Survey I water-seal spirometer (Collins Medical, Inc., Braintree, Massachusetts) and software from S&M Instruments (Doylestown, Pennsylvania), was used by technicians at all four recruitment centers. Stringent quality assurance procedures for spirometry testing exceeded ATS recommendations ([10](#_ENREF_10)).

In **FHS**, spirometry at the 5^th^, 6^th^, and 7^th^ Offspring Cohort examinations was performed using a Collins Survey II spirometer (Collins Medical, Inc., Braintree, MA), interfaced to pulmonary function data acquisition and quality control software (S and M Instruments, Doylestown, PA) and calibrated daily. Spirometry at the 8^th^ Offspring Cohort examination was performed using a Collins CPL system (nSpire Health Inc., Longmont, CO) which was calibrated daily. Spirometric maneuvers were performed according to contemporaneous ATS ([9](#_ENREF_9), [12](#_ENREF_12)) or European Respiratory Society (ERS)-ATS standards ([13](#_ENREF_13)).

Spirometry in **Health ABC** was completed at four time points (baseline, years 4, 7 and 9) in accordance with standardized guidelines of the ATS, as previously reported ([14](#_ENREF_14)). The study used a horizontal, dry rolling seal HF6 Spirometer (Sensor Medics Corporation, Yorba Linda, CA, USA) during clinical visits, and the EasyOne Model 2001 diagnostic spirometer (ndd Medizintechnik AG, Zurich, Switzerland) during home visits starting in year 8. The two devices were evaluated for comparability and provided virtually identical values. All FEV_1_ measures meeting the ATS criteria for acceptability were included in the current study.

The **KORA** studies (Cooperative Health Research in the Region of Augsburg) are a series of independent population based studies from the general population living in the region of Augsburg, southern Germany ([15](#_ENREF_15), [16](#_ENREF_16)). KORA F4 including 3,080 individuals was conducted from 2006-08. Baseline lung function tests were performed in random subsample of subjects born between 1946 and 1965 (age range 41-63 years, n = 1,321). Spirometry was performed in line with the ATS/ ERS recommendations ([13](#_ENREF_13), [17](#_ENREF_17)) using a pneumotachograph-type spirometer (Masterscreen PC, CardinalHealth, Würzburg, Germany) before and after inhalation of 200 μg salbutamol. The spirometer was calibrated daily using a calibration pump (CardinalHealth, Würzburg, Germany), and additionally, an internal control (examiner) was used to ensure constant instrumental conditions. Under the guidance of the experienced examiners at least 3 and at most 8 trials were recorded to obtain at least 2 acceptable and reproducible flow-volume curves. After completion of each test, the curves were visually inspected, maneuvers with artifacts excluded and results evaluated according to ATS/ERS recommendations [3]. The present study is based on maximum values of FEV_1_ measured before bronchodilation. On average 3.1 years later subjects were reexamined by spirometry (KORA F4L, n = 1,050, response rate 79.5%). Conditions for lung function measurements, including the examiners and the data evaluation, were the same in both cohorts. Both studies were approved by the Ethics Committee of the Bavarian Medical Association and informed consent was obtained from the study participants. Genotypes were available for 890 of those individuals.

In the **Lothian Birth Cohorts (LBC)** spirometry was completed at two time points; at 70 and 73 years of age in LBC1936 and at 79 and 87 years of age in LBC1921. For both cohorts a Micro Medical Spirometer was used, assessments were conducted sitting down without noseclips. The accuracy of the spirometer is ± 3% (following ATS recommendations in standardization of spirometry 1994 update for flows and volumes).

Spirometry was performed at two time-points (baseline and year 5) in the **Prospective Investigation of the Vasculature in Uppsala Seniors (PIVUS)** ([18](#_ENREF_18)). The study was performed in accordance with ATS recommendations ([9](#_ENREF_9)) using the α spirometer; Vitalograph Ltd; Buckingham, UK. The best value from three recordings was used. The Ethics Committee of the University of Uppsala approved the study, and the participants gave their informed consent.

The **Rotterdam Study (RS)** is a prospective population-based cohort study founded in 1990 in a suburb of Rotterdam, the Netherlands. The first cohort (RS I) consists of 7,983 participants, aged 55 years and over. Performing of spirometry was introduced in 2004. Spirometry was performed by trained paramedical personnel using a SpiroPro^®^ portable spirometer (Erich Jaeger, Hoechberg, Germany) and using a Carefusion MasterScreen PFT (as of 2009), according to ATS/ERS guidelines. Measurements were done at the visit of the Rotterdam Study’s research facility. FEV_1_, FVC and FEV_1_/FVC ratio were measured. All spirometry measures were validated by two researchers, of which one is a specialist in respiratory medicine, by assessment of all applicable flow-volume and flow-time curves.

Spirometry in **SAPALDIA** was completed at two time points 11 years apart. Identical spirometry devices and protocols were used at both examinations for SAPALDIA ([19](#_ENREF_19), [20](#_ENREF_20)) (Sensormedics model 2200, Yorba Linda, USA). At least three forced expiratory lung function maneuvers were performed by each participant and a minimum of two acceptable forced expiratory flows, FVC and FEV_1_ complying with ATS criteria were obtained ([9](#_ENREF_9)). Expiratory flow measures with the highest sum of FVC and FEV_1_ were taken from the same flow-volume curves to calculate the ratio of FEV_1_/FVC. No bronchodilator was administered. Participants were requested not to use beta-2-agonists or anticholinergic inhalers four hours prior to and long-acting beta agonists, oral beta-2-agonists, theophyline or oral antimuscarinic medication eight hours prior to the time of appointment of the examination.

Spirometry in **SHIP** was conducted using a variable pressure bodyplethysmograph equipped with a pneumotachograph (VIASYS Healthcare, MasterScreen Body/Diff., JAEGER, Hoechberg, Germany) which met the ATS criteria ([12](#_ENREF_12)). The procedures were conducted in a sitting position with subjects wearing a noseclip. The volume signal was calibrated with a 3.0 litre syringe connected to the pneumotachograph, in accordance with the manufacturer’s recommendations, and at least once daily. Barometric pressure, temperature and relative humidity were registered every morning. Volume calibration referred to ATP-conditions (Ambient Temperature Pressure) but resulting lung volumes were expressed as BTPS-corrected (Body Temperature Pressure Saturated) ([12](#_ENREF_12), [21](#_ENREF_21)). The tests were performed in accordance with ATS and ERS recommendations ([21](#_ENREF_21), [22](#_ENREF_22)) in the following order: 1) determination of static lung volumes, 2) forced spirometry.

Statistical analysis

In each cohort study, a linear mixed effects model was constructed to model the longitudinal trajectory of FEV_1_ (i.e. baseline FEV_1_ and its rate of change over time). A continuous time (slope) variable was included in the model to quantify the time (in years) elapsed between each FEV_1_ measurement point and the study baseline and the coefficient estimate for time conveys the rate of change in FEV_1_ over time (mL/year). To allow for variation in baseline FEV_1_ and the rate of change in FEV_1_ across participants, the intercept and time variable were specified as both fixed and random effects. This model is commonly used for repeated measurements over time on the same participant, and takes the simplistic form:

FEV_1ij_ = α­ + β*t_ij_ + a_i_ + b_i_*t_ij_ + e_ij_,

where α­ and β are fixed population effects for intercept and time (slope), respectively; a­_i_ and b_i_ are the individual intercept and slope for participant i; t_ij_ is the time value corresponding to the j^th^ FEV_1_ measurement for participant i; and FEV_1ij_ is the FEV_1_ value for participant i at the j^th^ measurement time; finally, e_ij_ represents the independent random error term and its values are assumed to be normally distributed (mean 0, variance σ^2^). It is assumed that the random effects, a_i_ and b_i_, are each independently, normally distributed with mean 0 and variance σ_a_^2^ and σ_b_^2^, respectively; a_i_ and b_i_ are not independent and their covariance is σ_ab_. Therefore, the mixed effects model explicitly estimates the covariate effects, random effects, the variance for each random effect, and the covariance between each pair of random effects. In addition, the mixed effects model provides flexibility in handling unbalanced longitudinal data, which is common in longitudinal cohort studies, and allows participants with only one FEV_1_ measurement to be included in the analysis as well as those with more than one measurement.

In each cohort study, a preliminary mixed effects model was constructed based on the above modeling framework and was additionally adjusted for the following covariates: gender, standing height at each time point, baseline age and baseline smoking pack-years, smoking status during study follow-up, the product term of smoking status x time, and study site and principal component variables for genetic ancestry as needed (family structure was accounted for where needed, for example in the Framingham Heart Study). Smoking status during study follow-up was defined as a four-level categorical variable as follows: never smokers at all time points (referent group), persistent smokers defined as current smokers at all time points, former smokers at all time points, and intermittent smokers defined as inconsistent smoker status across time points (switching between current and former status). The above main effects estimated how these covariates affected baseline FEV_1_ and the product term of smoking status x time estimated the effects of the three smoking statuses on the rate of change in FEV_1_ in comparison to never smokers.

Using the above preliminary model, each cohort study evaluated the presence and magnitude of covariance between the random intercept and time effects. When a meaningful covariance was present, the mixed effects model was specified to account for the covariance explicitly. Otherwise, the two random effects were specified as independent for model parsimony. Subsequently, residual diagnosis was performed in each cohort study based on the same preliminary model to exclude FEV_1_ measurements detected as outliers (i.e., |standardized residual| > 3 or 4, as determined by each study). Effect estimates of important covariates were compiled from all cohort studies for quality control and summary of overall patterns. The above preliminary modeling analyses were performed using Proc Mixed in SAS or lme4 or pedigreemm in R in each cohort study. Effect estimates for selected covariates across all cohort studies are presented in Table 1 and Supplementary Table E2.

For the final GWAS model, a SNP main effect and SNP x time interaction term were included in the above preliminary model; these terms estimated the effects of SNP genotype on baseline FEV_1_ and the rate of change in FEV_1_, respectively. All cohort studies performed the final GWAS analysis using either lme4 or pedigreemm (in FHS to account for familial correlation) in R. Study-specific results for both the SNP main and SNP x time interaction effects were shared and meta-analyses were performed using METAL software ([23](#_ENREF_23)) with the inverse variance weighting method to combine effect estimates after applying genomic control correction ([24](#_ENREF_24)).

Rationale for the meta-analysis of five cohort studies

There is noticeable phenotypic heterogeneity among the 14 cohort studies in the meta-analysis, as reflected by several important aspects of the longitudinal study design (Table 1). Two important factors related to the quality of the outcome measurement are the number of repeated measurements of lung function and the follow-up duration. Five cohort studies, namely BHS, CARDIA, CHS, FHS, and Health ABC, had three or more repeated measurements and relatively long follow-up durations in comparison to other studies, allowing for more precise estimation of the longitudinal trajectory in pulmonary function. This was supported empirically by the preliminary mixed effects model results, and the observation that these five cohort studies reported the smallest standard errors for the estimated rate of change in FEV_1_ in the reference group of never-smokers (Table 2). In light of this, we performed a second meta-analysis based on these five cohort studies with the goal to reduce noise due to phenotypic heterogeneity and thus gain improved statistical association signals.

SNP selection for follow-up analyses

The most statistically significant SNPs were first identified using a significance threshold of *P* ≤ 1 x 10^-5^ in either meta-analysis; all of the most statistically significant SNPs had a minor allele frequency above 5% (Tables E2 and E3). These SNPs were evaluated for regional linkage disequilibrium (LD), and multiple SNPs from a region were selected only when they met the p-value criterion and had an r^2^ estimate less than 0.7 with each other, according to HapMap CEU as implemented in SNAP ([25](#_ENREF_25)). Among SNPs in a given region with r^2^ values of 0.7 or greater with each other, the SNP with the lowest p-value was selected for follow-up analysis.

Expression Profiling

The mRNA expression profiles of the implicated genes at the novel chromosome 11 and 15 loci were determined using reverse transcription polymerase chain reaction (RT-PCR). Human airway smooth muscle (HASM) cells were isolated from healthy bronchial tissue of patients (with no previous history of asthma) undergoing surgery, and cultured as described previously ([26](#_ENREF_26)). Written consent was given by the patients and approval was granted by the Nottingham Local Ethical Research Committee (ref. EC00/165). Undifferentiated human bronchial epithelial cells (HBEC) (Lonza/Clonetics, Slough, UK) were maintained in culture as described previously ([27](#_ENREF_27)). Expression profiling by PCR used cDNA templates synthesised from total RNA, using the Superscript First-Strand Synthesis System for RT-PCR (Invitrogen/ Life Technologies Ltd., Paisley, UK), from cultured HASM and HBEC, as well as commercially available total lung and brain tissue (Ambion/Life Technologies Ltd.) and PBMC (3H Biomedical AB, Uppsala, Sweden). Primer sequences for the genes of interest are given in Table E7. PCR used the following cycling conditions: 35 cycles of 94°C for 1.5 minutes, 55°C for 1.5 minutes, and 72°C for 1.5 minutes, followed by 72°C for 10 minutes. Amplicons were extracted from agarose gels using the StrataPrep DNA Gel Extraction Kit (Agilent Technologies UK Ltd., Cheshire, UK) and validated by sequencing, using the BigDye Terminator v3.1 Cycle Sequencing Kit, in conjunction with an ABI PRISM 310 Genetic Analyser (Applied Biosystems/ Life Technologies Ltd.).

In addition, publicly available gene expression profiles of lung specimens from COPD patients (N=219) and controls (N=137) were obtained from the Lung Genomics Research Consortium (LGRC) site (<http://www.lung-genomics.org/>). Mean expression levels of the implicated genes at the novel chromosome 11 and 15 loci were compared between COPD patients and controls. The *P* value for the difference in means between the two groups was calculated using the two-sample t-test.

Finally, the most statistically significant SNPs at both loci (rs4077833 and rs507211, respectively) were tested for eQTL associations, using a publicly accessible eQTL database of lymphoblastoid cell lines ([28](#_ENREF_28)). A statistical significance threshold of *P* < 0.05 was used in both analyses.

**Table S1.** Details of SNP genotyping, quality control (QC), imputation, and statistical analysis across the 14 cohort studies

| Study | Genotyping platform | QC filters for excluding genotyped SNPs | N, genotyped autosomal SNPs passing QC | Imputation software | NCBI Build for imputation reference (HapMap CEU) | N, SNPs used for analysis (MAF>1%) | Statistical analysis software | Genomic control factor (λ_gc_) |
| --- | --- | --- | --- | --- | --- | --- | --- | --- |
| ARIC | Affymetrix 6.0 | call rate<95%,  HWE P<10^-6^,  MAF<1%, or  no chromosomal location | 669,450 | MACH v1.0.16 | build 36, release 22 | 2,449,419 | lme4, R | 1.04 |
| B58C^*^ | Illumina 550K (2 deposits) + 610K | call rate<95%,  HWE P<10^-4^,  MAF<1%, or  inconsistent (P<10^-4^) allele frequencies across 3 genotype deposits | 519,040 | MACH v1.0.16 | build 35, release 21 | 2,460,629 | lme4, R | 1.01 |
| BHS | \| Illumina 610-Quad \| \| --- \| | call rate<95%,  HWE P<5.7x10^-7^, or  MAF<1% | 549,294 | MACH v1.0.16 | build 36, release 22 | 2,420,960 | lme4, R | 1.05 |
| CARDIA | Affymetrix 6.0 | call rate<95%,  HWE P<10^-4^, or  MAF<2% | 578,568 | BEAGLE | build 36, release 22 | 2,276,434 | lme4, R | 1.03 |
| CHS | Illumina HumanHap 370CNV | call rate<97%,  no heterozygotes,  HWE P<10^-5^,  >2 duplicate errors,  Mendelian inconsistency  (for HapMap CEU trios), or  no mapping in dbSNP | 306,655 | BIMBAM | build 36, release 22 | 2,190,045 | lme4, R | 1.00 |
| FHS^†^ | Affymetrix 500K + 50K Human Gene Focused Panel | call rate<97%,  HWE P<10^-6^,  MAF<1%,  differential missingness related to genotype (mishap procedure in PLINK) with P<10^-9^,  Mendelian errors>100, or  absence from HapMap | 378,163 | MACH v1.0.15 | build 36, release 22 | 2,411,786 | pedigremm, R | 1.09 |
| Health ABC | Illumina Human1M-Duo | call rate < 95%,  HWE P<10^-6^, or  MAF > 1% | 914,263 | MACH | build 36, release 22 | 2,470,255 | lme4, R | 1.04 |
| KORA | Affymetrix 6.0 | call rate<93% | 909,622 | IMPUTE 0.4.2 | build 36, release 22 | 2,368,243 | lme4, R | 0.99 |
| LBC1921 | Ilumina 610-Quadv1 | call rate<98%,  HWE P<10^-3^, or  MAF<1% | 542,050 | MACH  v1.0.16 | build 36, release 22 | 2,302,855 | lme4, R | 0.97 |
| LBC1932 | Ilumina 610-Quadv1 | call rate<98%,  HWE P<10^-3^, or  MAF<1% | 542,050 | MACH  v1.0.16 | build 36, release 22 | 2,304,176 | lme4, R | 1.01 |
| PIVUS | Illumina OmniExpress + Metabochip | monomorphic, HWE P<10^-6^, call rate <95% if MAF>=5%, or call rate<99% if MAF<0.05 | 738,879 | IMPUTE 2.0 | build 36, release 22 | 2,436,058 | lme4, R | 1.00 |
| RS | Illumina HumanHap 550K | call rate<98%,  HWE P<10^-6^, or  MAF<1% | 537,405 | MACH v1.0.15 | build 36, release 22 | 2,377,064 | lme4, R | 0.96 |
| SAPALDIA | Illumina Human 610K quad | call rate<97%,  HWE P<10^-4^, or  MAF<5% | 582,892 | MACH v1.0.16 | build 36, release 22 | 2,456,064 | lme4, R | 1.11 |
| SHIP | Affymetrix 6.0 | none | 869,224 | IMPUTE v0.5.0 | build 36, release 22 | 2,450,720 | lme4, R | 0.99 |

*Definition of abbreviations*: ARIC = Atherosclerosis Risk in Communities; B58C = British 1958 Birth Cohort; BHS = Busselton Health Study; CARDIA = Coronary Artery Risk Development in Young Adults; CHS = Cardiovascular Health Study; FHS = Framingham Heart Study; Health ABC = Health, Aging, and Body Composition; HWE = Hardy Weinberg equilibrium; MAF = minor allele frequency; KORA = Cooperative Health Research in the Region of Augsburg; LBC1921 = Lothian Birth Cohort 1921; LBC1936 = Lothian Birth Cohort 1936; PIVUS = Prospective Investigation of the Vasculature in Uppsala Seniors; RS = Rotterdam Study; SAPALDIA = Swiss Study on Air Pollution and Lung Diseases in Adults; SHIP = Study of Health in Pomerania; SNP = single-nucleotide polymorphism.

^*^ Three original subsets of B58C were combined for this analysis, following a new phase of genotyping with a common platform.

^†^ To account for relatedness among subjects, the linear mixed effects regression model implemented in FHS used the pedigreemm package that adjusts for family structure ([29](#_ENREF_29)).

**Table S2.** Regression results for single nucleotide polymorphisms associated with the rate of change in FEV_1_ (mL/year) at *P* < 1 × 10^-5^ in the meta-analysis of 14 cohort studies (N=27,249)

| SNP | Chr | Position | Closest Gene(s) | Coded Allele | Noncoded Allele | Frequency | β | SE | *P* Value | Heterogeneity *P* Value |
| --- | --- | --- | --- | --- | --- | --- | --- | --- | --- | --- |
| rs12137475 | 1 | 44059735 | *ST3GAL3* | T | C | 0.11 | -3.52 | 0.76 | 3.90 × 10^-6^ | 0.48 |
| rs766488 | 1 | 61583103 | *NFIA* | A | G | 0.31 | 1.37 | 0.30 | 6.60 × 10^-6^ | 0.84 |
| rs17698444 | 1 | 215483178 | *ESRRG*  */GPATCH2* | C | G | 0.89 | -2.21 | 0.47 | 2.62 × 10^-6^ | 0.69 |
| rs12692550 | 2 | 159958017 | *BAZ2B* | T | C | 0.17 | -1.69 | 0.37 | 5.16 × 10^-6^ | 0.08 |
| rs2260732 | 13 | 113235802 | *TMCO3* | A | G | 0.28 | 1.42 | 0.31 | 4.83 × 10^-6^ | 0.25 |
| rs2260722 | 13 | 113236292 |  | A | G | 0.72 | -1.51 | 0.32 | 1.83 × 10^-6^ | 0.27 |
| rs2479753 | 13 | 113240886 |  | C | G | 0.28 | 1.42 | 0.31 | 4.77 × 10^-6^ | 0.22 |
| rs2259541 | 13 | 113253338 |  | C | G | 0.72 | -1.43 | 0.31 | 4.69 × 10^-6^ | 0.20 |
| rs3935740 | 15 | 79413780 | *IL16/STARD5*  */TMC3* | A | G | 0.09 | 2.23 | 0.49 | 4.54 × 10^-6^ | 1.00 |
| rs4077833 | 15 | 79419738 |  | C | G | 0.10 | 2.31 | 0.46 | 5.71 × 10^-7^ | 0.96 |
| rs8027498 | 15 | 89595638 | *SV2B* | A | G | 0.25 | 1.43 | 0.32 | 9.41 × 10^-6^ | 0.32 |
| rs8051319 | 16 | 15794449 | *MYH11* | T | C | 0.60 | 1.46 | 0.32 | 5.12 × 10^-6^ | 0.60 |
| rs740557 | 17 | 62451139 | *CACNG4* | C | G | 0.85 | -2.28 | 0.49 | 3.59 × 10^-6^ | 0.80 |

*Definition of abbreviations*: Chr = chromosome; SE = standard error; SNP = single-nucleotide polymorphism.

**Table S3.** Regression results for single nucleotide polymorphisms associated with the rate of change in FEV_1_ (mL/year) at *P* < 1 × 10^-5^ in the meta-analysis of the five cohort studies with three or more FEV_1_ measurements per participant (N=10,476)

| SNP | Chr | Position | Closest Gene(s) | Coded Allele | Noncoded Allele | Frequency | β | SE | *P* Value | Heterogeneity *P* Value |
| --- | --- | --- | --- | --- | --- | --- | --- | --- | --- | --- |
| rs10186544 | 2 | 28536678 | *FOSL2/PLB1* | T | C | 0.33 | 1.58 | 0.35 | 7.26 × 10^-6^ | 0.72 |
| rs10198727 | 2 | 28536753 |  | A | T | 0.67 | -1.58 | 0.35 | 7.45 × 10^-6^ | 0.71 |
| rs10209416 | 2 | 28536819 |  | A | G | 0.33 | 1.58 | 0.35 | 7.23 × 10^-6^ | 0.71 |
| rs10209501 | 2 | 28536881 |  | A | G | 0.33 | 1.58 | 0.35 | 7.09 × 10^-6^ | 0.71 |
| rs12692550 | 2 | 159958017 | *BAZ2B* | T | C | 0.18 | -2.01 | 0.42 | 2.02 × 10^-6^ | 0.02 |
| rs1729588 | 3 | 110790025 | *FLJ25363*  */MIR4445* | A | G | 0.30 | 1.60 | 0.36 | 8.38 × 10^-6^ | 0.33 |
| rs10764052 | 10 | 19863473 | *C10orf112* | T | C | 0.46 | 1.50 | 0.33 | 6.68 × 10^-6^ | 0.69 |
| rs10764053 | 10 | 19863644 |  | T | G | 0.47 | 1.53 | 0.33 | 4.15 × 10^-6^ | 0.76 |
| rs12219073 | 10 | 19870510 |  | C | G | 0.54 | -1.55 | 0.34 | 4.78 × 10^-6^ | 0.72 |
| rs17729837 | 10 | 19900824 |  | A | C | 0.52 | -1.50 | 0.33 | 6.21 × 10^-6^ | 0.69 |
| rs10740924 | 10 | 19907692 |  | C | G | 0.53 | 1.47 | 0.32 | 4.55 × 10^-6^ | 0.39 |
| rs7095285 | 10 | 19909284 |  | A | G | 0.53 | 1.47 | 0.32 | 4.23 × 10^-6^ | 0.39 |
| rs1409737 | 10 | 19926336 |  | A | G | 0.53 | 1.45 | 0.32 | 6.52 × 10^-6^ | 0.39 |
| rs12770750 | 10 | 19928004 |  | T | C | 0.47 | -1.45 | 0.32 | 6.38 × 10^-6^ | 0.45 |
| rs7898799 | 10 | 19929473 |  | T | C | 0.53 | 1.53 | 0.33 | 4.94 × 10^-6^ | 0.34 |
| rs10740927 | 10 | 19930342 |  | T | G | 0.47 | -1.45 | 0.32 | 6.36 × 10^-6^ | 0.45 |
| rs7915851 | 10 | 19933596 |  | A | G | 0.53 | 1.46 | 0.32 | 6.10 × 10^-6^ | 0.46 |
| rs627684 | 11 | 86050787 | *ME3* | A | G | 0.31 | 1.78 | 0.35 | 3.70 × 10^-7^ | 0.60 |
| rs601988 | 11 | 86050799 |  | A | G | 0.31 | 1.77 | 0.35 | 4.46 × 10^-7^ | 0.56 |
| rs507123 | 11 | 86054358 |  | A | G | 0.33 | 1.81 | 0.35 | 2.30 × 10^-7^ | 0.45 |
| rs507211 | 11 | 86054387 |  | A | G | 0.25 | 2.09 | 0.37 | 2.18 × 10^-8^ | 0.65 |
| rs626049 | 11 | 86054989 |  | T | C | 0.31 | 1.74 | 0.35 | 7.20 × 10^-7^ | 0.50 |
| rs594361 | 11 | 86064432 |  | A | G | 0.69 | -1.78 | 0.35 | 3.85 × 10^-7^ | 0.56 |
| rs642245 | 11 | 86067184 |  | A | G | 0.19 | 2.19 | 0.42 | 1.43 × 10^-7^ | 0.40 |

*Definition of abbreviations*: Chr = chromosome; SE = standard error; SNP = single-nucleotide polymorphism.**Table S4.** Association of the 14 sentinel SNPs from the meta-analyses in the AGES-Reykjavík study (AGES) and the Lung Health Study (LHS) for the rate of change in FEV_1_ (mL/year)

| SNP | Chr | Closest Gene(s) | Coded Allele | Freq | Meta-analysis^*^ | | | AGES  (n = 1,494) | | | LHS  (n = 4,048) | | |
| --- | --- | --- | --- | --- | --- | --- | --- | --- | --- | --- | --- | --- | --- |
|  |  |  |  |  | β | SE | *P* Value | β | SE | *P* Value | β | SE | *P* Value |
| Meta-analysis of 14 cohort studies (n = 27,249) | | | | | | | | | | | | | |
| rs12137475 | 1 | *ST3GAL3* | T | 0.11 | -3.5 | 0.8 | 3.90 × 10^-6^ | 1.2 | 1.5 | 0.42 | 2.6 | 2.5 | 0.30 |
| rs766488 | 1 | *NFIA* | A | 0.31 | 1.4 | 0.3 | 6.60 × 10^-6^ | 0.1 | 0.7 | 0.94 | -2.0 | 1.3 | 0.12 |
| rs17698444 | 1 | *ESRRG /GPATCH2* | C | 0.89 | -2.2 | 0.5 | 2.62 × 10^-6^ | -0.4 | 1.0 | 0.72 | -0.2 | 1.9 | 0.93 |
| rs12692550 | 2 | *BAZ2B* | T | 0.17 | -1.7 | 0.4 | 5.16 × 10^-6^ | 0.4 | 0.9 | 0.66 | -0.6 | 1.6 | 0.72 |
| rs2259541 | 13 | *TMCO3* | C | 0.72 | -1.5 | 0.3 | 1.83 × 10^-6^ | 0.8 | 0.7 | 0.29 | 0.0 | 1.3 | 0.99 |
| rs4077833 | 15 | *IL16/STARD5/TMC3* | C | 0.10 | 2.3 | 0.5 | 5.71 × 10^-7^ | -0.3 | 1.1 | 0.79 | -0.5 | 2.0 | 0.80 |
| rs8027498 | 15 | *SV2B* | A | 0.25 | 1.4 | 0.3 | 9.41 × 10^-6^ | 0.1 | 0.8 | 0.87 | -1.7 | 1.4 | 0.22 |
| rs8051319 | 16 | *MYH11* | T | 0.60 | 1.7 | 0.3 | 5.12 × 10^-6^ | -0.6 | 0.7 | 0.35 | 0.4 | 1.3 | 0.76 |
| rs740557 | 17 | *CACNG4* | C | 0.85 | -2.3 | 0.5 | 3.59 × 10^-6^ | -2.2 | 1.2 | 0.08 | 2.7 | 2.5 | 0.27 |
| Meta-analysis of the five cohort studies with three or more FEV_1_ measurements per participant (n = 10,476) | | | | | | | | | | | | | |
| rs10209501 | 2 | *FOSL2/PLB1* | A | 0.33 | 1.6 | 0.4 | 7.09 × 10^-6^ | -0.3 | 0.7 | 0.62 | -1.3 | 1.3 | 0.31 |
| rs12692550 | 2 | *BAZ2B* | T | 0.18 | -2.0 | 0.4 | 2.02 × 10^-6^ | 0.4 | 0.9 | 0.66 | -0.6 | 1.6 | 0.72 |
| rs1729588 | 3 | *FLJ25363 /MIR4445* | A | 0.30 | 1.6 | 0.4 | 8.38 × 10^-6^ | 0.9 | 0.7 | 0.20 | 0.9 | 1.3 | 0.48 |
| rs10764053 | 10 | *C10orf112* | A | 0.47 | 1.5 | 0.3 | 4.15 × 10^-6^ | 0.5 | 0.6 | 0.40 | -0.6 | 1.2 | 0.62 |
| rs507211 | 11 | *ME3* | A | 0.25 | 2.1 | 0.4 | 2.18 × 10^-8^ | 1.0 | 0.7 | 0.15 | 0.3 | 1.4 | 0.85 |

*Definition of abbreviations*: Chr = chromosome; Freq = frequency; SE = standard error; SNP = single-nucleotide polymorphism.

^*^ Association results of the 14 sentinel SNPs with the rate of change in FEV_1_ (mL/year) in the corresponding discovery meta-analysis are shown for comparison.

**Table S5.** Association of previously reported loci in GWAS of cross-sectional lung function ([1](#_ENREF_1), [30](#_ENREF_30), [31](#_ENREF_31)) with the rate of change in FEV_1_ (mL/year) in the meta-analysis of 14 cohort studies (N=27,249)

| SNP | Chr | Coded Allele | Frequency | β | SE | *P* Value^*^ | Gene | Reference |
| --- | --- | --- | --- | --- | --- | --- | --- | --- |
| Loci associated with cross-sectional FEV_1_ | | | | | | | | |
| rs2571445 | 2 | A | 0.39 | -0.18 | 0.29 | 0.54 | *TNS1* | 30 |
| rs1344555 | 3 | T | 0.20 | 0.24 | 0.36 | 0.52 | *MECOM* | 1 |
| rs17035960 | 4 | T | 0.07 | 0.05 | 0.57 | 0.93 | *GSTCD* | 31 |
| rs17036052 | 4 | T | 0.06 | 0.32 | 0.69 | 0.64 | *GSTCD* | 31 |
| rs17036090 | 4 | T | 0.93 | 0.09 | 0.57 | 0.88 | *GSTCD* | 31 |
| rs11727189 | 4 | T | 0.07 | -0.09 | 0.58 | 0.88 | *GSTCD* | 31 |
| rs10516526 | 4 | A | 0.93 | 0.07 | 0.55 | 0.90 | *GSTCD* | 30 |
| rs11097901 | 4 | T | 0.07 | -0.03 | 0.56 | 0.95 | *GSTCD* | 31 |
| rs11728716 | 4 | A | 0.07 | 0.05 | 0.55 | 0.92 | *GSTCD* | 31 |
| rs17036341 | 4 | C | 0.93 | -0.01 | 0.55 | 0.99 | *GSTCD* | 31 |
| rs17331332 | 4 | A | 0.08 | 0.07 | 0.55 | 0.90 | *GSTCD* | 31 |
| rs3995090 | 5 | A | 0.61 | 0.36 | 0.29 | 0.21 | *HTR4* | 30 |
| rs6889822 | 5 | A | 0.62 | 0.32 | 0.29 | 0.27 | *HTR4* | 30 |
| rs6903823 | 6 | A | 0.77 | 0.32 | 0.34 | 0.35 | *ZKSCAN3* | 1 |
| rs7068966 | 10 | T | 0.53 | -0.35 | 0.28 | 0.22 | *CDC123* | 1 |
| rs11001819 | 10 | A | 0.48 | -0.32 | 0.29 | 0.26 | *C10orf11* | 1 |
| Loci associated with cross-sectional FEV_1_/FVC | | | | | | | | |
| rs2284746 | 1 | C | 0.48 | -0.46 | 0.29 | 0.11 | *MFAP2* | 1 |
| rs993925 | 1 | T | 0.34 | 0.16 | 0.31 | 0.60 | *TGFB2* | 1 |
| rs10498230 | 2 | T | 0.07 | 1.59 | 0.57 | **0.005** | *PID1* | 31 |
| rs1435867 | 2 | T | 0.93 | -1.52 | 0.56 | **0.006** | *PID1* | 31 |
| rs12477314 | 2 | T | 0.21 | 0.04 | 0.36 | 0.90 | *HDAC4* | 1 |
| rs1529672 | 3 | A | 0.18 | 0.64 | 0.40 | 0.11 | *RARB* | 1 |
| rs6830970 | 4 | A | 0.65 | 0.16 | 0.30 | 0.60 | *FAM13A* | 31 |
| rs2869967 | 4 | T | 0.61 | 0.10 | 0.29 | 0.72 | *FAM13A* | 31 |
| rs1032295 | 4 | T | 0.59 | -0.55 | 0.30 | 0.07 | *HHIP* | 30 |
| rs12504628 | 4 | T | 0.60 | -0.48 | 0.29 | 0.10 | *HHIP* | 31 |
| rs1980057 | 4 | T | 0.40 | 0.57 | 0.29 | **0.048** | *HHIP* | 31 |
| rs153916 | 5 | T | 0.53 | -0.10 | 0.28 | 0.71 | *SPATA9* | 1 |
| rs11168048 | 5 | T | 0.58 | 0.26 | 0.29 | 0.38 | *HTR4* | 31 |
| rs7735184 | 5 | T | 0.40 | -0.30 | 0.29 | 0.29 | *HTR4* | 31 |
| rs2277027 | 5 | A | 0.65 | -0.37 | 0.29 | 0.20 | *ADAM19* | 31 |
| rs1422795 | 5 | T | 0.65 | -0.34 | 0.29 | 0.25 | *ADAM19* | 31 |
| rs2857595 | 6 | A | 0.19 | 0.37 | 0.38 | 0.33 | *NCR3* | 1 |
| rs2070600 | 6 | T | 0.05 | 0.64 | 0.68 | 0.34 | *AGER* | 30, 31 |
| rs2798641 | 6 | T | 0.19 | 0.13 | 0.37 | 0.72 | *ARMC2* | 1 |
| rs11155242 | 6 | A | 0.79 | -0.56 | 0.35 | 0.11 | *GPR126* | 31 |
| rs6937121 | 6 | T | 0.70 | -0.79 | 0.31 | **0.010** | *GPR126* | 31 |
| rs3817928 | 6 | A | 0.79 | -0.59 | 0.35 | 0.09 | *GPR126* | 31 |
| rs7776375 | 6 | A | 0.71 | -0.78 | 0.32 | **0.015** | *GPR126* | 31 |
| rs16909898 | 9 | A | 0.91 | -0.82 | 0.52 | 0.11 | *PTCH1* | 31 |
| rs10512249 | 9 | A | 0.09 | 0.73 | 0.50 | 0.14 | *PTCH1* | 31 |
| rs7068966 | 10 | T | 0.53 | -0.35 | 0.28 | 0.22 | *CDC123* | 1 |
| rs11172113 | 12 | T | 0.62 | -0.18 | 0.30 | 0.55 | *LRP1* | 1 |
| rs1036429 | 12 | T | 0.23 | -0.14 | 0.34 | 0.68 | *CCDC38* | 1 |
| rs12899618 | 15 | A | 0.15 | 0.61 | 0.40 | 0.13 | *THSD4* | 30 |
| rs12447804 | 16 | T | 0.20 | 0.02 | 0.36 | 0.95 | *MMP15* | 1 |
| rs2865531 | 16 | A | 0.59 | -0.58 | 0.29 | **0.042** | *CFDP1* | 1 |
| rs9978142 | 21 | A | 0.85 | -0.42 | 0.41 | 0.31 | *KCNE2* | 1 |

*Definition of abbreviations*: Chr = chromosome; SE = standard error; SNP = single-nucleotide polymorphism.

^*^ *P* values below the statistically significant threshold of 0.05 are shown in bold.

**Table S6.** mRNA expression profiling of the implicated genes at the two novel loci in human lung and control tissues^*^

| Gene | Human Tissues/Cells | | | | |
| --- | --- | --- | --- | --- | --- |
|  | Lung | Human Airway Smooth Muscle Cells | Human Bronchial Epithelial Cells | Peripheral Blood Mononuclear Cells | Brain |
| *IL16* | + | + | - | + | + |
| *STARD5* | + | + | + | + | + |
| *TMC3* | - | - | - | - | + |
| *ME3* | + | + | + | - | + |

^*^ Primer sequences are provided in Table E7. A “+” sign indicates the presence of the transcript, and a “-” sign indicates its absence. All products were sequence verified.

**Table S7.** Primers for mRNA expression profiling

| **Gene** | **Sequence** |
| --- | --- |
| ***IL16*** | Forward primer 5’-CTCGCTCAACCTTTCAGAGC-3’ |
|  | Reverse primer 5’-TCTGTGAACCGTAATCACCTTG-3’ |
| ***STARD5*** | Forward primer 5’-AGGGAACCTGTACCGAGGAG-3’ |
|  | Reverse primer 5’-GGTGGGTTCCCCTGGAAG-3’ |
| ***TMC3*** | Forward primer 5’-CATTCCAGAGCTGATTGCAG-3’ |
|  | Reverse primer 5’-GGTAGCCATTTCCTCAATGC-3’ |
| ***ME3*** | Forward primer 5’-GACCTGGACAAGTACATCATTCTC-3’ |
|  | Reverse primer 5’-TGGCAGCAACACCTATGATG-3’ |

**Table S8.** Summary of eQTL look-up for the most significant SNPs at the novel chromosome 11 and 15 loci

| SNP (location) | eQTL Type | Associated Gene | Chr | *P* Value | Gene Function |
| --- | --- | --- | --- | --- | --- |
| rs4077833  (*IL16/STARD5/TMC3* locus on chr 15) | Trans | *NR1I2* | 3 | 6.84 × 10^-4^ | Nuclear receptor subfamily 1, group I, member 2 (*NR1I2*) encodes a pleiotropic nuclear transcription factor with a key role in the regulation of *CYP3A4*, a cytochrome P450 enzyme that metabolizes more than 50% of human clinical drugs ([32](#_ENREF_32)). *NR1I2* is activated by a range of endogenous and xenobiotic compounds and binds to response elements in the promoter regions of many target genes in complex with the retinoic acid receptor RXR. |
| rs507211  (*ME3* locus on chr 11) | Trans | *KIAA1109* | 4 | 5.20 × 10^-4^ | *KIAA1109* is part of a linkage disequilibrium block (*KIAA1109-TENR-IL2-IL21* gene cluster) associated with susceptibility to celiac disease ([33](#_ENREF_33)); this region encodes the interleukins IL2 and IL21. In mice, IL21 levels are higher in blood and lung tissues of animals exposed to cigarette smoke vs. air-exposed ([34](#_ENREF_34)). Murine *in vitro* work shows that IL21 promotes T-cell induced apoptosis and cell damage, suggesting a possible link between gene expression in this region and lung outcomes ([34](#_ENREF_34)). Additionally, in mammals KIAA1109 is thought to function in the regulation of epithelial growth and differentiation, and in tumor development ([35](#_ENREF_35)). |

*Definition of abbreviations*: Chr = chromosome; eQTL = expression quantitative trait loci; SNP = single-nucleotide polymorphism.

**Figure S1.** Manhattan and QQ plots for the meta-analysis of the rate of change in FEV_1_ in 14 cohort studies


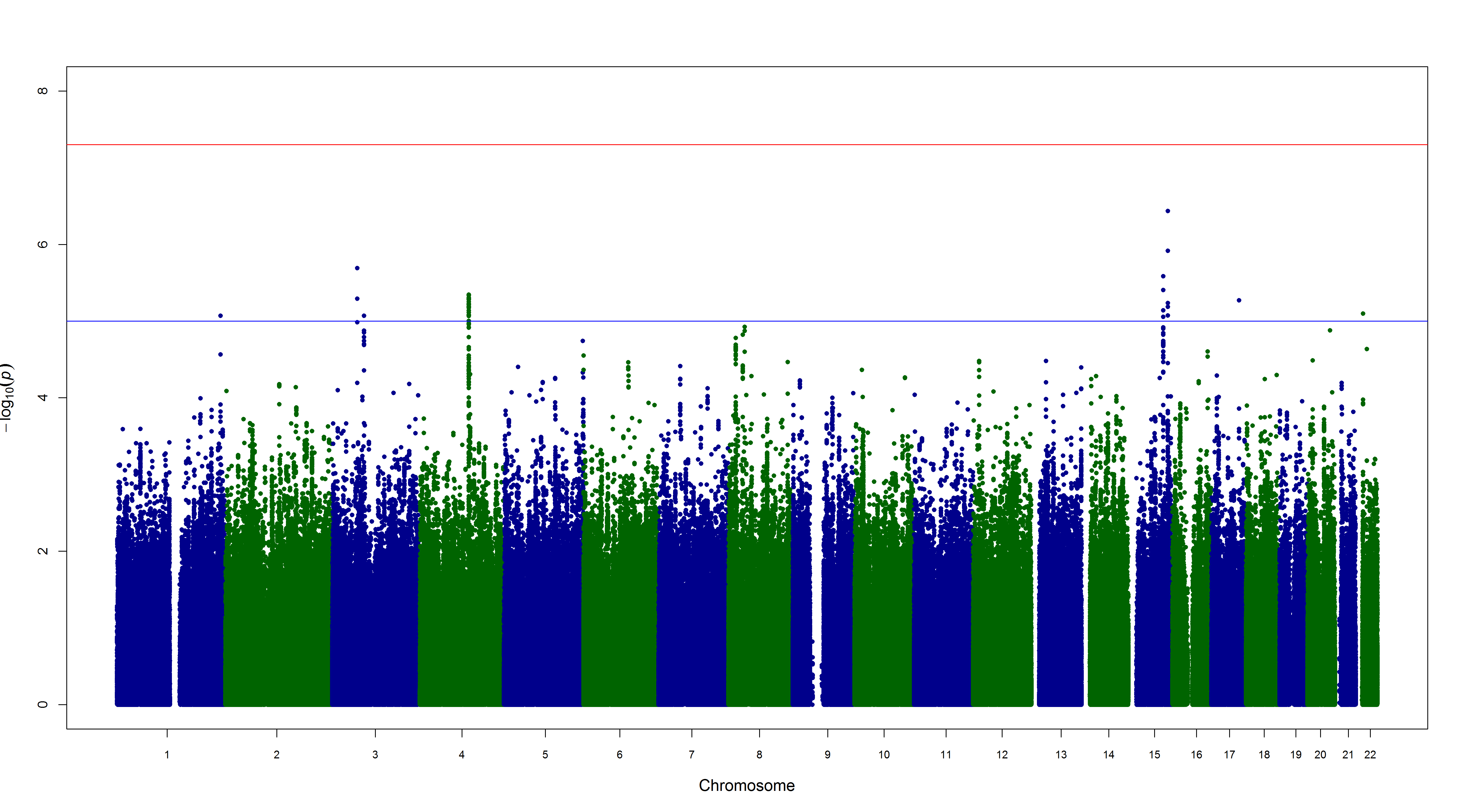


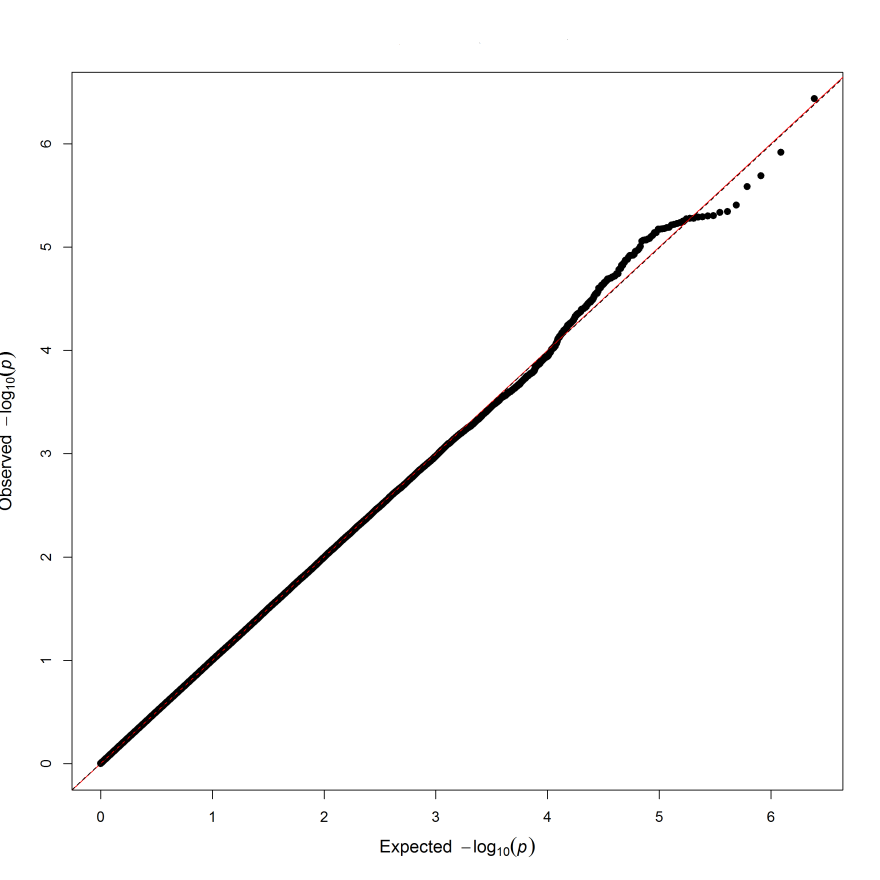


**Figure S2.** Manhattan and QQ plots for the meta-analysis of the rate of change in FEV_1_ in the five cohort studies with three or more FEV_1_ measurements per participant


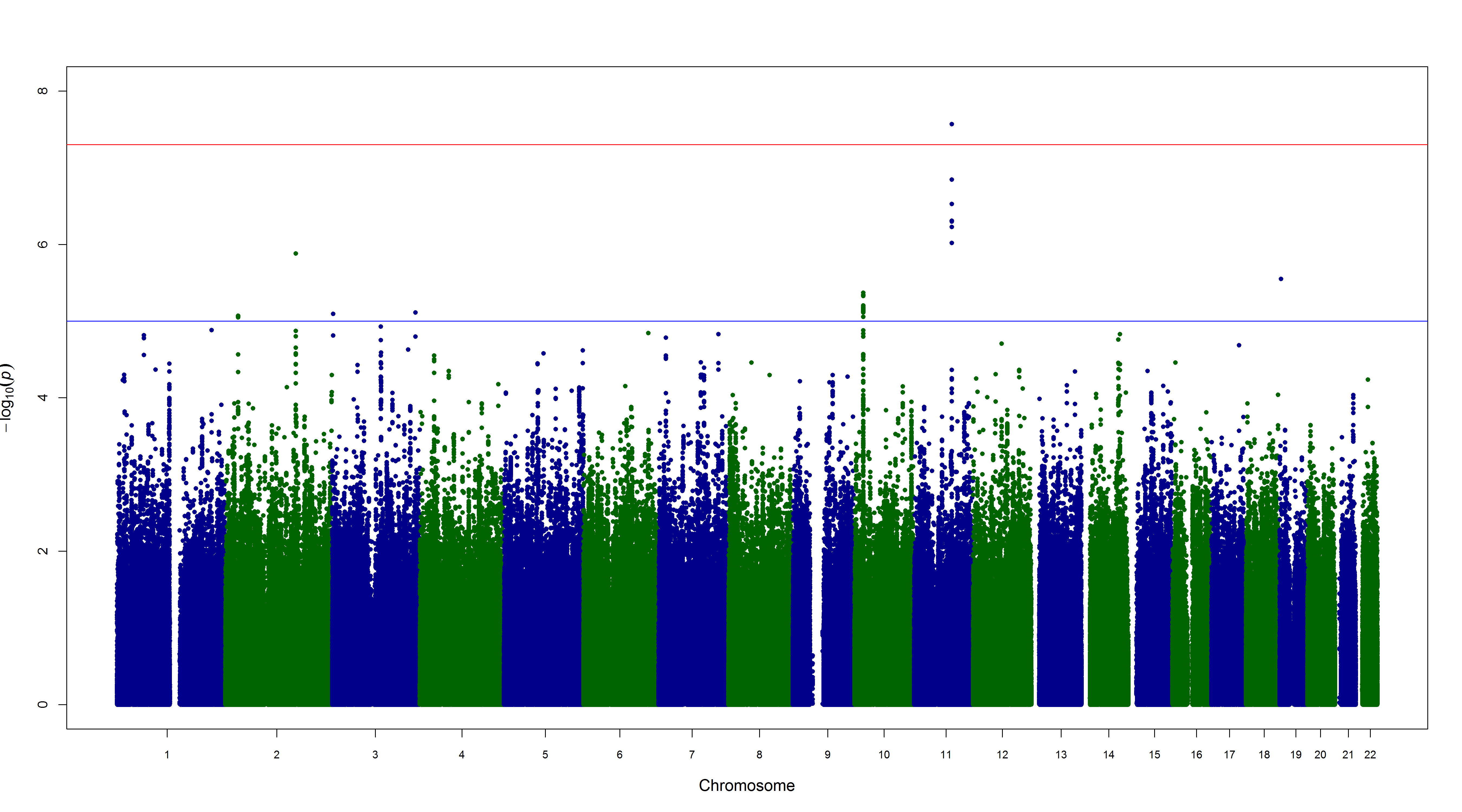


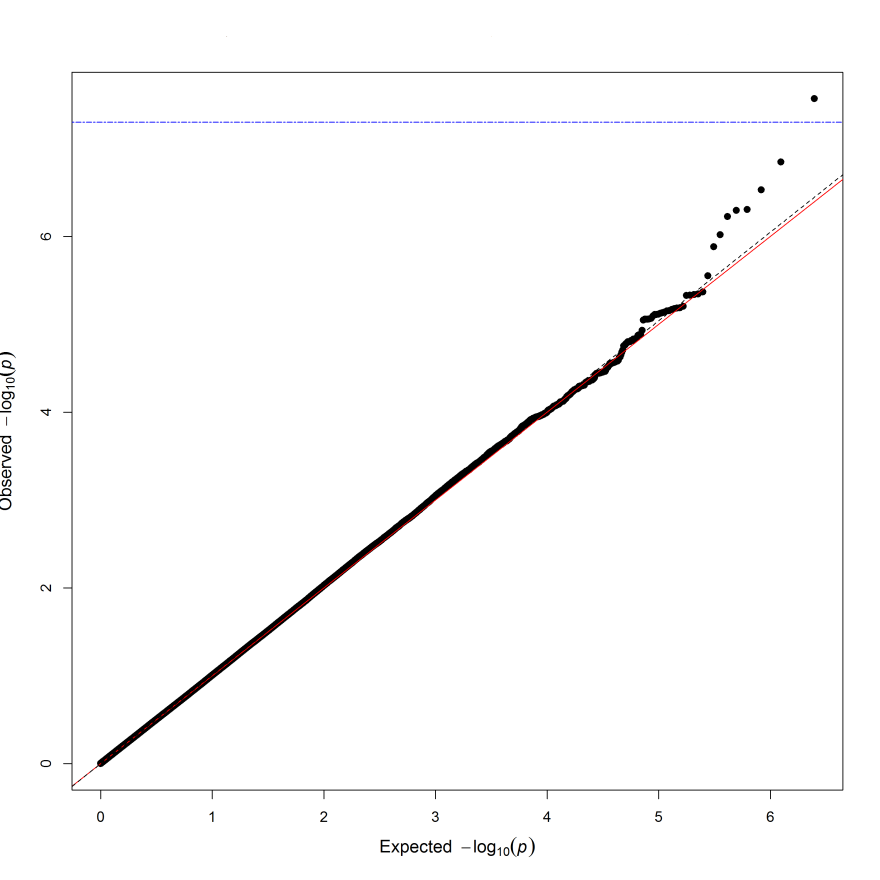


**Figure S3.** mRNA expression profiling in human lung samples from 219 COPD patients and 137 controls for A) *IL16*, B) *STARD5*, and C) *ME3*, using publicly available microarray data from the Lung Genomics Research Consortium site (<http://www.lung-genomics.org/>). The y-axes reflect the probe intensities of each gene transcript in the binary logarithm form, with the red dots indicating the average probe intensities and the red bars indicating standard deviation. The *P* values were calculated using the two-sample t-test.

A. *IL16*


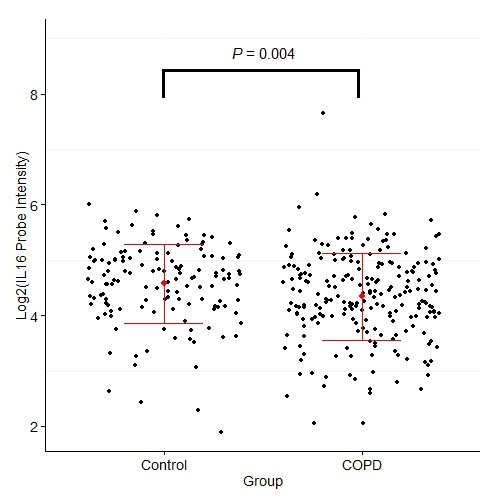


B. *STARD5*


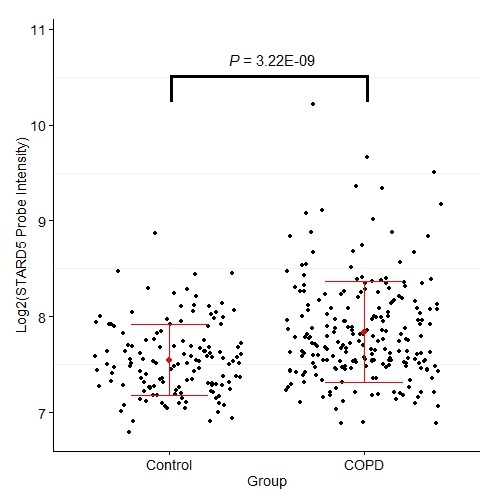


C. *ME3*


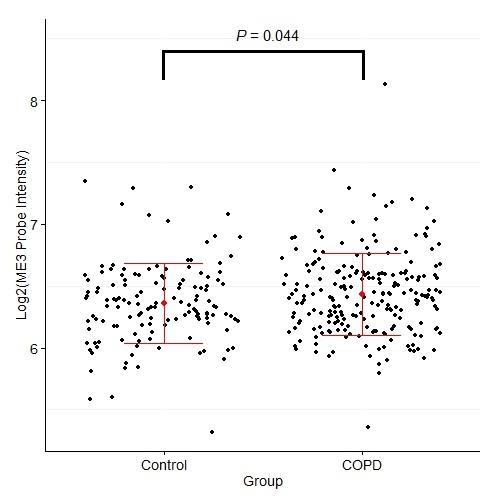


**References:**

1. Soler Artigas M, Loth DW, Wain LV, Gharib SA, Obeidat M, Tang W, Zhai G, Zhao JH, Smith AV, Huffman JE, Albrecht E, Jackson CM, Evans DM, Cadby G, Fornage M, Manichaikul A, Lopez LM, Johnson T, Aldrich MC, Aspelund T, Barroso I, Campbell H, Cassano PA, Couper DJ, Eiriksdottir G, Franceschini N, Garcia M, Gieger C, Gislason GK, Grkovic I, Hammond CJ, Hancock DB, Harris TB, Ramasamy A, Heckbert SR, Heliovaara M, Homuth G, Hysi PG, James AL, Jankovic S, Joubert BR, Karrasch S, Klopp N, Koch B, Kritchevsky SB, Launer LJ, Liu Y, Loehr LR, Lohman K, Loos RJ, Lumley T, Al Balushi KA, Ang WQ, Barr RG, Beilby J, Blakey JD, Boban M, Boraska V, Brisman J, Britton JR, Brusselle GG, Cooper C, Curjuric I, Dahgam S, Deary IJ, Ebrahim S, Eijgelsheim M, Francks C, Gaysina D, Granell R, Gu X, Hankinson JL, Hardy R, Harris SE, Henderson J, Henry A, Hingorani AD, Hofman A, Holt PG, Hui J, Hunter ML, Imboden M, Jameson KA, Kerr SM, Kolcic I, Kronenberg F, Liu JZ, Marchini J, McKeever T, Morris AD, Olin AC, Porteous DJ, Postma DS, Rich SS, Ring SM, Rivadeneira F, Rochat T, Sayer AA, Sayers I, Sly PD, Smith GD, Sood A, Starr JM, Uitterlinden AG, Vonk JM, Wannamethee SG, Whincup PH, Wijmenga C, Williams OD, Wong A, Mangino M, Marciante KD, McArdle WL, Meibohm B, Morrison AC, North KE, Omenaas E, Palmer LJ, Pietilainen KH, Pin I, Pola Sbreve Ek O, Pouta A, Psaty BM, Hartikainen AL, Rantanen T, Ripatti S, Rotter JI, Rudan I, Rudnicka AR, Schulz H, Shin SY, Spector TD, Surakka I, Vitart V, Volzke H, Wareham NJ, Warrington NM, Wichmann HE, Wild SH, Wilk JB, Wjst M, Wright AF, Zgaga L, Zemunik T, Pennell CE, Nyberg F, Kuh D, Holloway JW, Boezen HM, Lawlor DA, Morris RW, Probst-Hensch N, Kaprio J, Wilson JF, Hayward C, Kahonen M, Heinrich J, Musk AW, Jarvis DL, Glaser S, Jarvelin MR, Ch Stricker BH, Elliott P, O'Connor GT, Strachan DP, London SJ, Hall IP, Gudnason V, Tobin MD. Genome-wide association and large-scale follow up identifies 16 new loci influencing lung function. *Nat Genet* 2011;43:1082-1090.

2. The atherosclerosis risk in communities (ARIC) study: Design and objectives. The ARIC investigators. *Am J Epidemiol* 1989;129:687-702.

3. Strachan DP, Griffiths JM, Johnston ID, Anderson HR. Ventilatory function in british adults after asthma or wheezing illness at ages 0-35. *Am J Respir Crit Care Med* 1996;154:1629-1635.

4. Strachan DP, Rudnicka AR, Power C, Shepherd P, Fuller E, Davis A, Gibb I, Kumari M, Rumley A, Macfarlane GJ, Rahi J, Rodgers B, Stansfeld S. Lifecourse influences on health among british adults: Effects of region of residence in childhood and adulthood. *Int J Epidemiol* 2007;36:522-531.

5. Marossy AE, Strachan DP, Rudnicka AR, Anderson HR. Childhood chest illness and the rate of decline of adult lung function between ages 35 and 45 years. *Am J Respir Crit Care Med* 2007;175:355-359.

6. James AL, Palmer LJ, Kicic E, Maxwell PS, Lagan SE, Ryan GF, Musk AW. Decline in lung function in the busselton health study: The effects of asthma and cigarette smoking. *Am J Respir Crit Care Med* 2005;171:109-114.

7. Ryan G, Knuiman MW, Divitini ML, James A, Musk AW, Bartholomew HC. Decline in lung function and mortality: The busselton health study. *J Epidemiol Community Health* 1999;53:230-234.

8. ATS statement--snowbird workshop on standardization of spirometry. *Am Rev Respir Dis* 1979;119:831-838.

9. Standardization of spirometry, 1994 update. American thoracic society. *Am J Respir Crit Care Med* 1995;152:1107-1136.

10. Enright PL, Kronmal RA, Higgins M, Schenker M, Haponik EF. Spirometry reference values for women and men 65 to 85 years of age. Cardiovascular health study. *Am Rev Respir Dis* 1993;147:125-133.

11. Enright PL, Kronmal RA, Higgins MW, Schenker MB, Haponik EF. Prevalence and correlates of respiratory symptoms and disease in the elderly. Cardiovascular health study. *Chest* 1994;106:827-834.

12. Standardization of spirometry--1987 update. Statement of the american thoracic society. *Am Rev Respir Dis* 1987;136:1285-1298.

13. Miller MR, Hankinson J, Brusasco V, Burgos F, Casaburi R, Coates A, Crapo R, Enright P, van der Grinten CP, Gustafsson P, Jensen R, Johnson DC, MacIntyre N, McKay R, Navajas D, Pedersen OF, Pellegrino R, Viegi G, Wanger J. Standardisation of spirometry. *Eur Respir J* 2005;26:319-338.

14. Waterer GW, Wan JY, Kritchevsky SB, Wunderink RG, Satterfield S, Bauer DC, Newman AB, Taaffe DR, Jensen RL, Crapo RO. Airflow limitation is underrecognized in well-functioning older people. *J Am Geriatr Soc* 2001;49:1032-1038.

15. Holle R, Happich M, Lowel H, Wichmann HE. Kora--a research platform for population based health research. *Gesundheitswesen* 2005;67 Suppl 1:S19-25.

16. Wichmann HE, Gieger C, Illig T. Kora-gen--resource for population genetics, controls and a broad spectrum of disease phenotypes. *Gesundheitswesen* 2005;67 Suppl 1:S26-30.

17. Karrasch S, Flexeder C, Behr J, Holle R, Huber RM, Jorres RA, Nowak D, Peters A, Wichmann HE, Heinrich J, Schulz H. Spirometric reference values for advanced age from a south german population. *Respiration; international review of thoracic diseases* 2013;85:210-219.

18. Lind L, Fors N, Hall J, Marttala K, Stenborg A. A comparison of three different methods to evaluate endothelium-dependent vasodilation in the elderly: The prospective investigation of the vasculature in uppsala seniors (pivus) study. *Arteriosclerosis, thrombosis, and vascular biology* 2005;25:2368-2375.

19. Kunzli N, Ackermann-Liebrich U, Keller R, Perruchoud AP, Schindler C. Variability of fvc and fev1 due to technician, team, device and subject in an eight centre study: Three quality control studies in sapaldia. Swiss study on air pollution and lung disease in adults. *Eur Respir J* 1995;8:371-376.

20. Kunzli N, Kuna-Dibbert B, Keidel D, Keller R, Brandli O, Schindler C, Schweinzer KM, Leuenberger P, Ackermann-Liebrich U. Longitudinal validity of spirometers--a challenge in longitudinal studies. *Swiss medical weekly* 2005;135:503-508.

21. Nelson SB, Gardner RM, Crapo RO, Jensen RL. Performance evaluation of contemporary spirometers. *Chest* 1990;97:288-297.

22. Wanger J, Clausen JL, Coates A, Pedersen OF, Brusasco V, Burgos F, Casaburi R, Crapo R, Enright P, van der Grinten CP, Gustafsson P, Hankinson J, Jensen R, Johnson D, Macintyre N, McKay R, Miller MR, Navajas D, Pellegrino R, Viegi G. Standardisation of the measurement of lung volumes. *Eur Respir J* 2005;26:511-522.

23. Willer CJ, Li Y, Abecasis GR. Metal: Fast and efficient meta-analysis of genomewide association scans. *Bioinformatics* 2010;26:2190-2191.

24. Devlin B, Roeder K. Genomic control for association studies. *Biometrics* 1999;55:997-1004.

25. Johnson AD, Handsaker RE, Pulit SL, Nizzari MM, O'Donnell CJ, de Bakker PI. Snap: A web-based tool for identification and annotation of proxy snps using hapmap. *Bioinformatics* 2008;24:2938-2939.

26. Daykin K, Widdop S, Hall IP. Control of histamine induced inositol phospholipid hydrolysis in cultured human tracheal smooth muscle cells. *Eur J Pharmacol* 1993;246:135-140.

27. Danahay H, Atherton H, Jones G, Bridges RJ, Poll CT. Interleukin-13 induces a hypersecretory ion transport phenotype in human bronchial epithelial cells. *Am J Physiol Lung Cell Mol Physiol* 2002;282:L226-236.

28. Dixon AL, Liang L, Moffatt MF, Chen W, Heath S, Wong KC, Taylor J, Burnett E, Gut I, Farrall M, Lathrop GM, Abecasis GR, Cookson WO. A genome-wide association study of global gene expression. *Nat Genet* 2007;39:1202-1207.

29. Vazquez AI, Bates DM, Rosa GJ, Gianola D, Weigel KA. Technical note: An r package for fitting generalized linear mixed models in animal breeding. *Journal of animal science* 2010;88:497-504.

30. Repapi E, Sayers I, Wain LV, Burton PR, Johnson T, Obeidat Me, Zhao JH, Ramasamy A, Zhai G, Vitart V, Huffman JE, Igl W, Albrecht E, Deloukas P, Henderson J, Granell R, McArdle WL, Rudnicka AR, Barroso I, Loos RJF, Wareham NJ, Mustelin L, Rantanen T, Surakka I, Imboden M, Wichmann HE, Grkovic I, Jankovic S, Zgaga L, Hartikainen A-L, Peltonen L, Gyllensten U, Johansson Å, Zaboli G, Campbell H, Wild SH, Wilson JF, Gläser S, Homuth G, Völzke H, Mangino M, Soranzo N, Spector TD, Polašek O, Rudan I, Wright AF, Heliövaara M, Ripatti S, Pouta A, Naluai ÅT, Olin A-C, Torén K, Cooper MN, James AL, Palmer LJ, Hingorani AD, Wannamethee SG, Whincup PH, Smith GD, Ebrahim S, McKeever TM, Pavord ID, MacLeod AK, Morris AD, Porteous DJ, Cooper C, Dennison E, Shaheen S, Karrasch S, Schnabel E, Schulz H, Grallert H, Bouatia-Naji N, Delplanque J, Froguel P, Blakey JD, Britton JR, Morris RW, Holloway JW, Lawlor DA, Hui J, Nyberg F, Jarvelin M-R, Jackson C, Kähönen M, Kaprio J, Probst-Hensch NM, Koch B, Hayward C, Evans DM, Elliott P, Strachan DP, Hall IP, Tobin MD. Genome-wide association study identifies five loci associated with lung function. *Nat Genet* 2009;42:36-44.

31. Hancock DB, Eijgelsheim M, Wilk JB, Gharib SA, Loehr LR, Marciante KD, Franceschini N, van Durme YMTA, Chen T-h, Barr RG, Schabath MB, Couper DJ, Brusselle GG, Psaty BM, van Duijn CM, Rotter JI, Uitterlinden AG, Hofman A, Punjabi NM, Rivadeneira F, Morrison AC, Enright PL, North KE, Heckbert SR, Lumley T, Stricker BHC, O'Connor GT, London SJ. Meta-analyses of genome-wide association studies identify multiple loci associated with pulmonary function. *Nat Genet* 2009;42:45-52.

32. Orans J, Teotico DG, Redinbo MR. The nuclear xenobiotic receptor pregnane x receptor: Recent insights and new challenges. *Mol Endocrinol* 2005;19:2891-2900.

33. van Heel DA, Franke L, Hunt KA, Gwilliam R, Zhernakova A, Inouye M, Wapenaar MC, Barnardo MC, Bethel G, Holmes GK, Feighery C, Jewell D, Kelleher D, Kumar P, Travis S, Walters JR, Sanders DS, Howdle P, Swift J, Playford RJ, McLaren WM, Mearin ML, Mulder CJ, McManus R, McGinnis R, Cardon LR, Deloukas P, Wijmenga C. A genome-wide association study for celiac disease identifies risk variants in the region harboring il2 and il21. *Nat Genet* 2007;39:827-829.

34. Duan MC, Huang Y, Zhong XN, Tang HJ. Th17 cell enhances cd8 t-cell cytotoxicity via il-21 production in emphysema mice. *Mediators of inflammation* 2012;2012:898053.

35. Kuo MT, Wei Y, Yang X, Tatebe S, Liu J, Troncoso P, Sahin A, Ro JY, Hamilton SR, Savaraj N. Association of fragile site-associated (fsa) gene expression with epithelial differentiation and tumor development. *Biochem Biophys Res Commun* 2006;340:887-893.
